# Hybrid Self‐Assembled Gel Beads for Tuneable pH‐Controlled Rosuvastatin Delivery

**DOI:** 10.1002/chem.202101405

**Published:** 2021-08-19

**Authors:** Carmen C. Piras, Anna K. Patterson, David K. Smith

**Affiliations:** ^1^ Department of Chemistry University of York Heslington, York YO10 5DD UK

**Keywords:** drug delivery, gels, self-assembly, statins, supramolecular chemistry

## Abstract

This article describes the fabrication of new pH‐responsive hybrid gel beads combining the polymer gelator calcium alginate with two different low‐molecular‐weight gelators (LMWGs) based on 1,3 : 2,4‐dibenzylidene‐d‐sorbitol: pH‐responsive DBS‐COOH and thermally responsive DBS‐CONHNH_2_, thus clearly demonstrating that different classes of LMWG can be fabricated into gel beads by using this approach. We also demonstrate that self‐assembled multicomponent gel beads can be formed by using different combinations of these gelators. The different gel bead formulations exhibit different responsiveness – the DBS‐COOH network can disassemble within those beads in which it is present upon raising the pH. To exemplify preliminary data for a potential application for these hybrid gel beads, we explored aspects of the delivery of the lipid‐lowering active pharmaceutical ingredient (API) rosuvastatin. The release profile of this statin from the hybrid gel beads is pH‐dependent, with greater release at pH 7.4 than at pH 4.0 – primary control of this process results from the p*K*_a_ of the API. The extent of pH‐mediated API release is also significantly further modified according to gel bead composition. The DBS‐COOH/alginate beads show rapid, highly effective drug release at pH 7.4, whereas the three‐component DBS‐COOH/DBS‐CONHNH_2_/alginate system shows controlled slow release of the API under the same conditions. These initial results indicate that such gel beads constitute a promising, versatile and easily tuned platform suitable for further development for controlled drug‐delivery applications.

## Graphical abstract

**It′s all in the delivery**: Self‐assembled gel beads have been fabricated using different supramolecular gelators in combination with a calcium alginate polymer gelator and loaded with the statin drug rosuvastatin. The rate of rosuvastatin release is shown to depend on both the pH of the receiving solution and the precise gel bead composition. As such, these gel beads are capable of doubly controlled release.

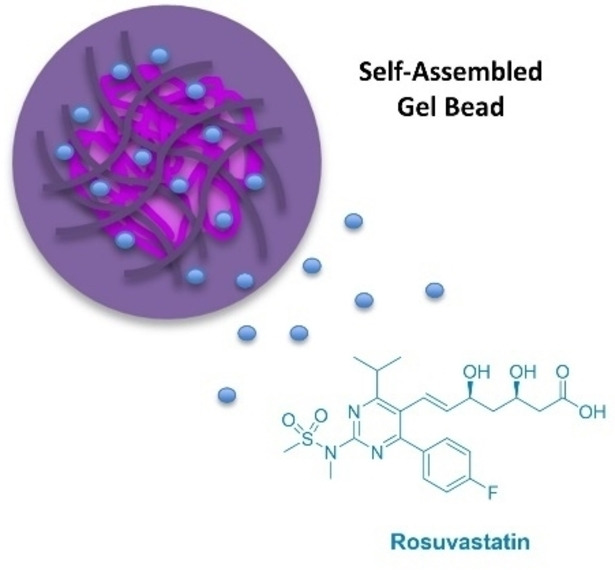

## Introduction

Hydrogels self‐assembled from low‐molecular‐weight gelators (LMWGs) are versatile, multifunctional materials of intense current interest due to their responsive and tuneable nature.^[1]^ In particular, there has been considerable recent development of such materials for uses in biomedicine, drug delivery and tissue regeneration.^[2]^ In drug delivery, hydrogels can potentially release active pharmaceutical ingredients (API) at a target site in response to specific stimuli, such as pH changes. Being robust, easy‐to‐prepare and handle, hydrogels formed from polymer gelators (PGs) have been widely employed as drug delivery materials.^[3]^ However, LMWGs are gaining increasing attention as an alternative to polymers for next‐generation therapeutic carriers, as a result of advantages offered by their synthetic programmability, biodegradability and high degree of responsiveness to external stimuli.^[2]^

Compared to polymeric materials, hydrogels assembled from LMWGs are often soft and difficult to handle without breakage. As such, LMWGs have often been considered as particularly attractive candidates for transdermal or subcutaneous injection delivery modes.^[4]^ However, there is increasing interest in the development of more robust shaped and patterned formulations of LMWGs for a range of applications.^[5]^ The formulation of shaped and structured LMWG‐based systems, such as capsules or beads, remains a fascinating challenge.^[6]^ Such systems have potential both in oral drug delivery for controlled release in the GI tract, or in the development of implantable or injectable drug delivery depots, which could achieve either controlled local API delivery, or slow longer‐term systemic API release.

**Figure 1:**
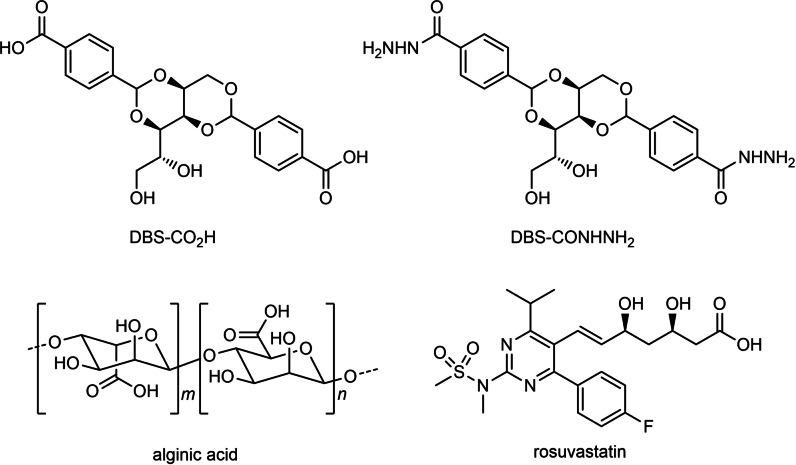
Chemical structures of the LMWGs DBS‐COOH and DBS‐CONHNH_2_, the PG component alginic acid and the statin drug rosuvastatin.

The combination of LMWGs with PGs to obtain hybrid gels that display greater robustness, whilst maintaining responsiveness to external stimuli is a known strategy to expand the range of potential applications of such materials.^[7]^ In this regard, we recently reported a hybrid gel based on the LMWG 1,3 : 2,4‐di(4‐acylhydrazide)‐benzylidene‐d‐sorbitol^.^(DBS‐CONHNH_2_) and the natural polysaccharide PG calcium alginate (Figure 1).^[8]^ As a result of the orthogonal gelation mechanisms, this combination could be formulated into core‐shell gel beads. By loading these gel beads with metal nanoparticles, we demonstrated their potential both in catalysis^[8a]^ and antibacterial^[8b]^ applications. We have also recently used this approach to generate smaller injectable microgel beads with diameters of about 800 nm, and shown that such microgels can release bioactive agents to enhance tissue growth.^[9]^ With the goal of controlled drug delivery, we have previously been interested in multicomponent hydrogels.^[10]^ As a result, we decided to develop gel beads with stimulus responsiveness. We reasoned that incorporating the pH‐responsive gelator DBS‐COOH, and drawing on our previous experience of combining it with DBS‐CONHNH_2_,^[11]^ could be a way of achieving this. Importantly, both of these LMWGs have been previously demonstrated to be biocompatible as part of multicomponent systems used for tissue growth.^[12]^ We herein describe the preparation of pH‐responsive self‐assembled gel beads obtained by combining these LMWGs with calcium alginate, the first time a pH‐responsive LMWG has been combined with an alginate PG, and subsequently explore, in a preliminary way, their potential for the release of the lipid‐lowering medication rosuvastatin.^[13]^

Statins are key cholesterol‐lowering medications widely prescribed to the general public as a preventative against adverse events such as heart attacks and strokes.^[12]^ As one example, rosuvastatin (Figure 1) is usually administered orally as a tablet in a 5–40 mg daily dose and is mainly absorbed from the intestine (pH 8.0).^[14]^ Statins have also been suggested to have positive effects on bone growth^[15]^ and are being particularly explored in the field of dentistry for the treatment of periodontitis.^[16]^ There has therefore been considerable interest in the local delivery of statins, including their formulation with simple polymer gels.^[17]^

Given the need for both local and systemic delivery of this important class of drug, there is considerable ongoing interest in alternative statin formulations. For example, enhanced GI delivery may better deliver some of these poorly soluble drugs, while injectable or implantable delivery systems would also be of value either in local, or long‐term statin delivery. It is worth noting that the relatively low daily dose of drugs (<40 mg/day) makes them particularly well suited to this approach, as it facilitates drug loading into the gel. Amongst this class of drug, we were particularly interested in rosuvastatin as it is acid‐functionalised, and as demonstrated in our previous work with the painkiller anti‐inflammatory drug naproxen,^[10]^ we reasoned this should introduce a degree of inherent pH control into the delivery process. To the best of our knowledge, this is the first example of an LMWG being used to control the release of a statin drug.

## Results and Discussion

### DBS‐COOH/alginate gel beads

**Figure 2:**
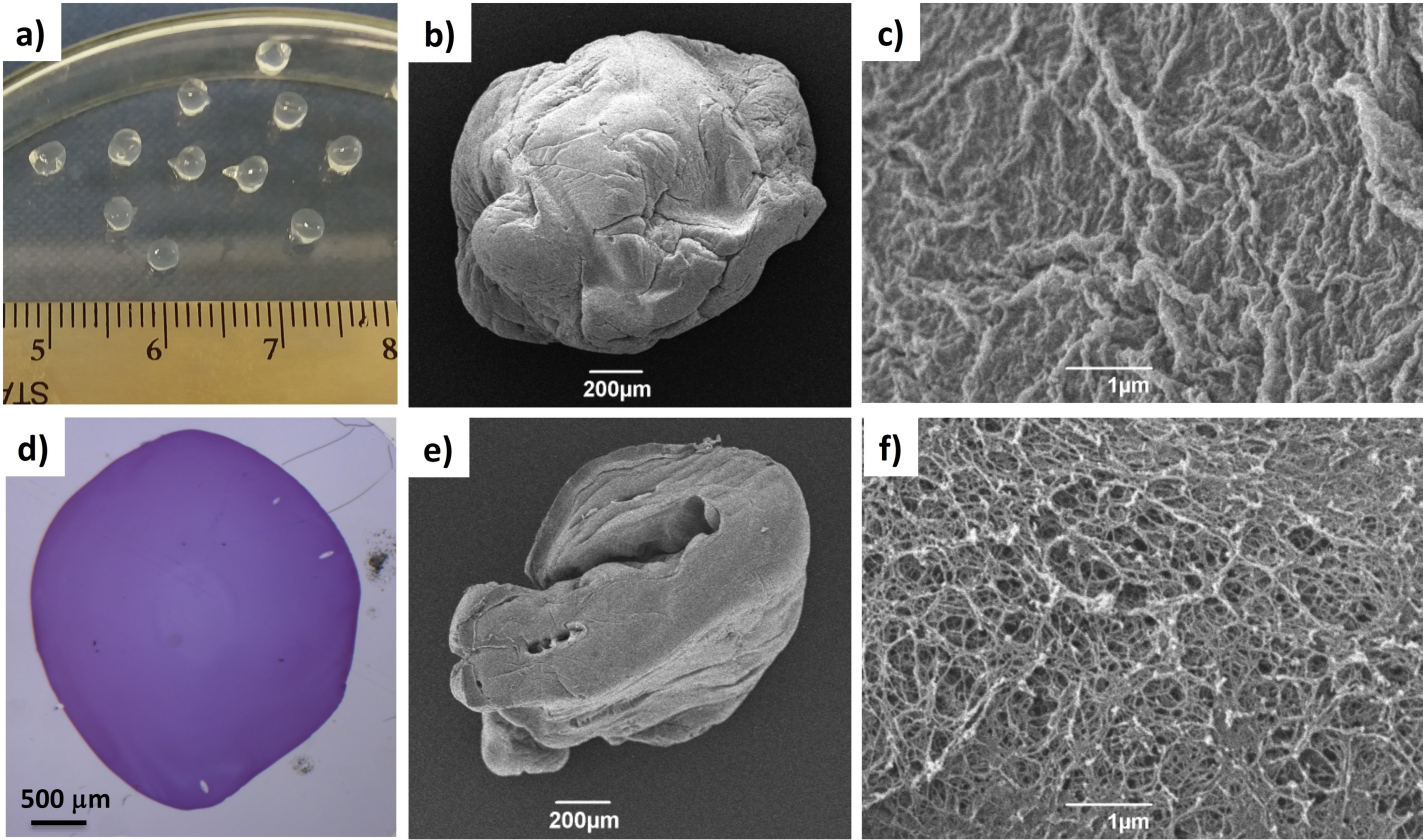
a) DBS‐COOH/alginate gel beads. SEM images of b) a whole gel bead and c) the gel bead surface. d) Optical microscopy of a DBS‐COOH/alginate gel bead cross‐section embedded into resin and stained with toluidine blue. e) and f) SEM images of DBS‐COOH/alginate gel bead cross‐section at different magnifications.

DBS‐COOH was synthesized in good yield using our previously reported method.^[18]^ This LMWG forms hydrogels at low concentrations (0.2–0.3 % *w*/*v*) in a mildly acidic pH regime below its p*K*_a_ value of 5.4. Alginate is a commercially available PG that forms hydrogels when combined with bivalent cations, such as Ca^2+^.^[19]^ Since these two gelation triggers can be temporally controlled, we decided to fabricate two‐component DBS‐COOH/alginate hydrogels by inducing the self‐assembly of the PG first, followed by slower pH‐induced assembly of the LMWG. To form gel beads, we therefore combined a basic DBS‐COOH aqueous solution (0.3 % *w*/*v*) with sodium alginate (0.5 % *w*/*v*). The resulting mixture was added dropwise (20 μL drops) to a CaCl_2_ solution (5.0 % *w*/*v*), which had been acidified by addition of HCl to give an 0.01 M concentration of acid. Small gel beads were immediately formed (Figure 2a and Figure S13 in the Supporting Information). The gel beads were initially transparent and then turned more translucent, which would suggest a process in which, as predicted, the PG assembled first, and then the LMWG assembled. Indeed, in its own right, when treated in sample vials in this way, the LMWG forms a translucent self‐assembled gel. These gel beads had diameters of 2.8–3.3 mm (Figure 2a, b and d), which relates to the 20 μL drop volume. Beads with different diameters could be obtained by adding different volumes to the cross‐linker solution (Figure S13). Importantly, this is the first time a pH‐responsive LMWG has been combined with calcium alginate, and demonstrates the versatility of the alginate LMWG stabilization methodology previously developed in our labs.^[8]^ In addition to forming gel beads, we also formed hybrid gels in vials for detailed characterisation and control experiments – these systems are described fully in the Supporting Information.

As gelation of the two networks depended on both the H^+^ and Ca^2+^ ions in the acidic CaCl_2_ solution, we reasoned that the two self‐assembled networks would be woven within the whole gel bead volume, rather than forming core‐shell structures, as previously described for the DBS‐CONHNH_2_/alginate gel beads. This hypothesis was confirmed by optical microscopy of the gel beads cut in half, embedded in resin and dyed with toluidine blue, which showed a uniform hybrid gel bead cross‐section comparable to that of beads formed by calcium alginate alone (Figures 2d and S14). SEM provided an insight into the fibrous structures of the surface and cross‐section of the gel beads (Figure 2c, e and f). The DBS‐COOH/alginate gel bead surface appeared to be wrinkled and densely packed (Figures 2c, S22 and S23). The cross‐section image indicated an extended network throughout the gel bead, confirming that the gelators were in their self‐assembled state inside the bead (Figures 2e, f and S25). We saw no evidence by SEM of precipitated, unstructured material within the beads, consistent with the view that the LMWG was indeed self‐assembling under controlled conditions during bead fabrication.

To further confirm the self‐assembly of DBS‐COOH within the gel bead, we performed a simple ^1^H NMR experiment. We prepared ten DBS‐COOH/alginate beads using D_2_O instead of water and transferred them into a NMR tube containing D_2_O (0.5 mL) and MeCN (3.0 μL) as an internal standard (Section S3.1 in the Supporting Information, ). If the DBS‐COOH was not in its self‐assembled state, the percentage of the mobile components could be calculated by comparison of the integral peaks of the DBS‐COOH aromatic peaks (*δ*=7.55 and 7.95) to that of the methyl group of acetonitrile (*δ*=2.09 ppm). The ^1^H NMR spectrum showed no signals for either DBS‐COOH or alginate (Figure S1), thus confirming that both gelators were fully immobilised within the gel beads. Combined with the results from imaging, this clearly indicates that controlled self‐assembly of the LMWG occurs.

The exact amount of DBS‐COOH incorporated into each gel bead was then quantified, also by ^1^H NMR (Section S3.2). The DBS‐COOH/alginate gel beads were prepared in water (1 mL) using 0.3 % *w*/*v* DBS‐COOH (6.72 μmol) and 0.5 % *w*/*v* alginate. Ten gel beads were isolated and dried under vacuum. The resulting solid was dissolved in [D_6_]DMSO, which dissolved the DBS‐COOH, but not the alginate, and a known volume of MeCN (3.0 μL) was added as an internal standard. The concentration of DBS‐COOH was calculated by comparison of the integrals of the DBS‐COOH aromatic peaks (*δ*=7.55 and 7.95) to that of the methyl group of acetonitrile (*δ*=2.09 ppm). The experiment showed that each gel bead incorporated 0.13 μmol of DBS‐COOH (Figure S2). Considering that the gel beads were prepared using a 20 μL volume, in principle 50 gel beads could be obtained from the 1 mL of solution used. If each gel bead contained 0.13 μmol of DBS‐COOH, as demonstrated by NMR spectroscopy, 50 gel beads would contain a total of 6.34 μmol of DBS‐COOH, which corresponds to about 94 % of the DBS‐COOH initially loaded. This reproducible experiment therefore confirms the efficiency of the fabrication method with almost all of the added DBS‐COOH ending up assembled within the gel beads.

To verify the responsiveness of the DBS‐COOH/alginate gel beads to pH, we performed a further ^1^H NMR study. Ten gel beads were prepared in D_2_O and transferred into a NMR tube. D_2_O (0.5 mL), NaOD (0.5 M, 60 μL, ca. 23 equiv.) and the internal standard MeCN (3.0 μL) were then added (Section S3.3). The sample was left undisturbed overnight and the ^1^H NMR spectrum was recorded at 30 min intervals. The percentage of mobile components was calculated by comparison of the integrals of the aromatic peaks of the DBS‐COOH to that of acetonitrile (Figure S3). As the pH rises to about 10.8–11.6, the DBS‐COOH network is disrupted and disassembles (Figure S4). This process occurs rapidly, and indeed after just 30 minutes, about 40 % of the DBS‐COOH incorporated into each gel bead becomes mobile. On monitoring for extended periods this does not rise any further, indicating the rapid response of these gel beads to pH change. It is possible that the remaining DBS‐COOH (60 %) interacts with the calcium alginate network, preventing its full mobility (see Discussion below).

The supramolecular interactions between the LMWG and the PG within the self‐assembled gel beads were investigated by IR spectroscopy. A clear shift of the O−H stretch of alginate to lower wavenumber is visible in the presence of the DBS‐COOH network for the hybrid gel (Figure S10). The C=O stretch of DBS‐COOH also shifted to lower frequencies in the presence of increasing alginate concentrations (Figure S10). These shifts suggest some supramolecular interactions between the two gel networks in the hybrid gel bead, and are similar to what was observed for the corresponding interpenetrated gels (Figure S9). This might also help explain why about 50 % of the DBS‐COOH did not become mobile when the pH of the system was raised. We suggest that some of the DBS‐COOH may remain associated with the calcium alginate network under basic conditions – it is plausible that the carboxylate ion will interact with the divalent calcium ions present within the system.

We performed parallel plate rheology on equivalent two‐component gels formed in vials, to demonstrate that the presence of the calcium alginate network enhances the mechanical strength and stiffness of these gels (Table S2, Figures S26, S32–S34 and S36). Specifically, the elastic modulus (G’) of the DBS‐COOH gel (0.4 % *w*/*v*) progressively increases from 360 Pa to 905, 2660, 5300 and 12000 Pa with increasing alginate loadings of 0.1, 0.3, 0.5 and 1.0 % *w*/*v*, respectively.

In summary, we therefore confirmed that these two gelators form co‐assembled gel beads with the interwoven networks behaving in similar ways to within a bulk gel, and that the DBS‐COOH network could be individually addressed within these gels by raising the pH.

### DBS‐CONHNH_2_/alginate gel beads

Beads based on DBS‐CONHNH_2_ and calcium alginate were prepared and characterised as previously described,^[8]^ in which a hot aqueous solution of DBS‐CONHNH_2_ is added dropwise into a CaCl_2_ solution. This yields beads with a core‐shell structure. All characterisation of these beads was in‐line with our previous work (see also Figures S16 and S37).

### DBS‐COOH/DBS‐CONHNH_2_/alginate gel beads

**Figure 3:**
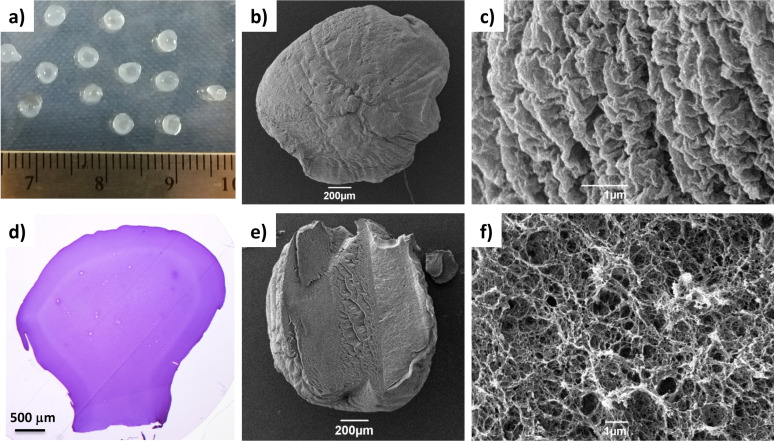
a) DBS‐COOH/DBS‐CONHNH_2_/alginate gel beads. SEM images of b) a whole gel bead and c) the gel bead surface. d) Optical microscopy of a DBS‐COOH/DBS‐CONHNH_2_/alginate gel bead cross‐section embedded into resin and stained with toluidine blue. e) and f) SEM images of DBS‐COOH/DBS‐CONHNH_2_/alginate gel bead cross‐sections.

We then combined all three gelators to obtain multicomponent gel beads – this level of complexity in LMWG gel beads has not previously been achieved and it is therefore important to carefully characterise this multicomponent system.^[20]^ We combined the procedures for the two different types of gel bead, and mixed a basic DBS‐COOH aqueous solution (0.3 % *w*/*v*) with DBS‐CONHNH_2_ (0.3 % *w*/*v*) and sodium alginate (0.5 % *w*/*v*). The resulting suspension was sonicated to help the dispersion of the solid particles and then heated until complete dissolution of the DBS‐CONHNH_2_. The hot solution was then added dropwise (20 μL drops) to a CaCl_2_ solution (5.0 % *w*/*v*), acidified with HCl at a concentration of 0.01 M. Small gel beads were immediately formed on simultaneous self‐assembly of the three gel networks (Figure 3a). As expected based on the 20 μL drop volume, they had diameters of 3.0–3.5 mm (Figure 3a, b and d). The diameter could be tuned by changing the volume added to the crosslinker solution.

We reasoned that these gel beads may have a core‐shell structure as a result of the use of a hot solution, similar to DBS‐CONHNH_2_/alginate beads. We suggest the core may contain the self‐assembled DBS‐CONHNH_2_/DBS‐COOH, wrapped in a DBS‐COOH/alginate shell. The hypothesis of a core‐shell nature was confirmed this by optical microscopy of a cross‐section of the gel beads embedded in resin and stained with blue toluidine (Figures 3d and S17). The surface and cross‐section of the gel beads were analysed by SEM microscopy which showed that the bead surface was wrinkled (Figure 3c and S24) and indicated the presence of a fibrous network inside the beads (Figure 3e, f and S25), suggesting that the gelators were incorporated into the gel beads in their self‐assembled state. Once again there was no evidence of precipitated, unstructured LMWG material within the beads.

The self‐assembly of all three gelators was further confirmed by a simple ^1^H NMR experiment performed on ten gel beads prepared in D_2_O, using MeCN as an internal standard, in the same way as described above for the DBS‐COOH/alginate gel beads (Section S3.4). The absence of signals in the spectrum demonstrated that the three gelators were in their self‐assembled state (Figure S4). The amount of DBS‐COOH and DBS‐CONHNH_2_ incorporated into the multicomponent gel beads was quantified by ^1^H NMR, once again as described above for the DBS‐COOH/alginate gel beads (Section S3.5). Ten DBS‐COOH/DBS‐CONHNH_2_/alginate gel beads were dried under vacuum and the solid was dissolved in [D_6_]DMSO. By comparing the integrals of the aromatic protons of DBS‐COOH and DBS‐CONHNH_2_ (DBS‐COOH aromatic peak *δ*=7.95 and DBS‐CONHNH_2_ aromatic peak *δ* =7.82) to that of the methyl group of the internal standard MeCN (*δ*=2.09), we were able to calculate that 0.13 μmol of DBS‐COOH and 0.11 μmol of DBS‐CONHNH_2_ were incorporated into each gel bead (Figure S6). This corresponds to >90 % of each of the loaded LMWGs, confirming the efficiency of the preparation method.

We performed parallel plate rheology on equivalent two‐component gels formed in vials, to demonstrate that the presence of the calcium alginate network enhances the mechanical strength and stiffness (Table S2, Figures S39–S42, S44). Specifically, the DBS‐COOH/DBS‐CONHNH_2_ gel prepared using a 0.3 % *w*/*v* concentration of the two LMWGs had an elastic modulus of 5410 Pa. In the presence of increasing alginate concentrations (0.1, 0.3, 0.5 and 1.0 % *w*/*v*), the *G*’ value increased to 6 950, 15 200, 19 000 and 37 300 Pa, respectively. These gels were stiffer than the DBS‐COOH/DBS‐CONHNH_2_ gel, reflecting the additional stiffness provided by the calcium alginate. Furthermore, they were significantly stiffer than the DBS‐COOH/alginate gel described above, indicating that this three‐component gel system assembles a denser, more interactive network (see below).

To verify the responsiveness of these three‐component DBS‐COOH/DBS‐CONHNH_2_/alginate gel beads to pH, we performed another ^1^H NMR study, as previously described for the two‐component DBS‐COOH/alginate system. Ten gel beads were prepared in D_2_O and transferred into a NMR tube. D_2_O (0.5 μL), NaOD (0.5 M, 60 μL, ca. 23 equiv.) and the internal standard MeCN (3.0 μL) were then added (Section S3.6) giving a pH value of 10.8–11.6. The sample was left undisturbed overnight and the ^1^H NMR spectrum was then recorded every 30 min. The percentage of mobile components was calculated by comparison of the integrals of the aromatic peaks of the two LMWGs to that of acetonitrile (Figures S7 and S8). It was found that about 50 % of the DBS‐COOH was rapidly mobilised in the first 30 minutes within the beads. This figure rose to about 60 % after 2.5 h and then remained approximately constant. This treatment also caused a minor effect on the DBS‐CONHNH_2_ with about 25 % of the DBS‐CONHNH_2_ being mobilised in the first 30 min, rising to 30 % after 1 h, and then remaining approximately constant. This may reflect disruption of the supramolecular interactions between the two LMWGs within the gel beads‐it is known that when these gelators are co‐assembled there are some interactions between the sequentially formed gel networks.^[11]^

Furthermore, we used rheology to investigate the pH‐responsiveness of DBS‐COOH/DBS‐CONHNH_2_/alginate gels in vials, and demonstrated that the *G*’ value dropped from 37 300 to 28 300 Pa after exposure to a small amount of aqueous NaOH for 24 h (Figure S36), raising the pH to 10.8–11.6. After further exposure to glucono‐δ‐lactone as a proton source (lowering the pH to ca. 4.0) the *G*’ value increased again to 34 500 Pa (Figure S37), indicating that not only can the DBS‐COOH network be disassembled and re‐assembled in these gels, but that this process has a direct impact on the rheological performance of the hybrid gels, with the stiffness decreasing as the DBS‐COOH network disassembles and increasing as it reassembles again.

### Applications of gel beads in rosuvastatin release

Taking into account the pH‐responsiveness of these gel bead formulations, we decided to focus on drug delivery to exemplify a potential application. Rosuvastatin displays a characteristic UV absorbance peak at 251 nm, therefore drug release from the gels in different pH buffers could be monitored by UV‐vis spectroscopy at regular time intervals. We selected two different release media: a neutral pH buffer, 10 mM Tris⋅HCl/150 mM NaCl (pH 7.4), and a mildly acidic pH buffer, 0.1 M sodium acetate (pH 4.0). To make sure that the observed UV signal was produced by the rosuvastatin released from the gels rather than disruption of the self‐assembled gel networks, negative control experiments on gels without rosuvastatin were carried out at the same time and the collected data were normalized accordingly. In general, the amount of LMWG released from the gel beads was only very small (see the Supporting Information).

We considered loading the drug during gel bead fabrication, and note that in some cases this may actually be the most appropriate and desirable approach. However, we were concerned that in our workflow for bead preparation, the drug may simply leach from the beads during stirring in the large volume aqueous Ca^2+^/H^+^ bath. This would lead to difficulties quantifying the amount of drug incorporated into the beads. As such, for this initial study, we decided to load the drug after the formation of the gel beads using a “soaking” methodology, which allowed effective quantification of drug loading. We anticipated that this loading method initially relies on simple diffusion, but that specific interactions between the drug and the gel networks would then have the chance to establish themselves as loading progressed.

To understand the loading process further, the gels in vials were loaded with rosuvastatin in the same way, and then studied using parallel plate rheology. As a general rule, the rosuvastatin‐loaded gels were broadly similar to those with no API present (Table S2, Figures S30, S35, S38 and S43). This indicates that the presence of the API does not significantly impact on the assembled gel network. Analysing the data in more detail suggested that in the presence of the API, most of the gels became slightly less stiff. The one exception was DBS‐CONHNH_2_/alginate loaded with rosuvastatin, where the stiffness increased slightly – this might reflect noncovalent interactions between the acid‐functionalised rosuvastatin and the DBS‐CONHNH_2_ gel network (see Discussion) leading to the slight network stiffening.

All gel beads used in the drug release studies were therefore prepared as described above and subsequently loaded with rosuvastatin calcium by soaking in an aqueous solution of the drug (0.11 mM, 4 mL). After 24 h, the drug solution was removed and used to quantify the exact amount of drug incorporated into each gel by UV‐vis spectroscopy (ca. 0.22 μmol/gel bead). The release buffer (6 mL) was then added to the gel samples, which were then incubated at 37 °C for 24 h. Drug release was then monitored at regular time intervals by UV‐vis spectroscopy (251 nm).

**Figure 4:**
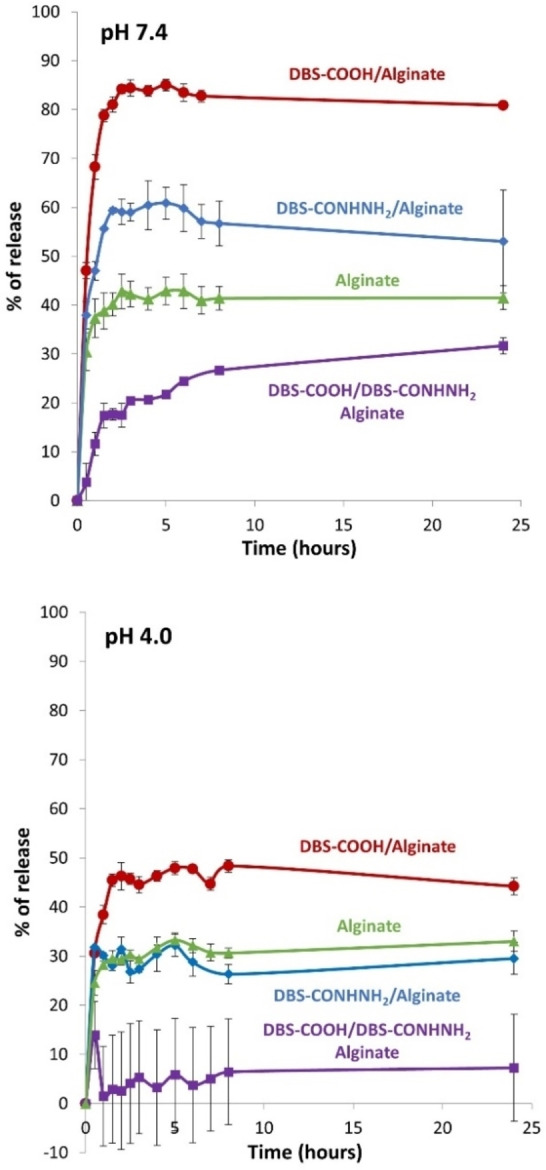
Rosuvastatin calcium % released in top: 10 mM Tris ⋅ HCl/150 mM NaCl (pH 7.4) from gel beads and bottom: in 0.1 M sodium acetate (pH 4.0) from gel beads.

Initially, we explored the release of rosuvastatin calcium into 10 mM Tris ⋅ HCl/150 mM NaCl at pH 7.4 (Figure 4, top, Table S4). For the DBS‐COOH/alginate gel beads, about 85 % release was achieved after 24 h, with release being mostly complete (80 %) after about 2 h. Leaving the system for 1 week eventually gave 85–90 % release. In the DBS‐CONHNH_2_/alginate gel beads release was about 55–60 % after 24 h (65 % after 1 week), and for calcium alginate beads was about 40 % after 24 h (45 % after 1 week). For the three‐component DBS‐COOH/DBS‐CONHNH_2_/alginate system, API release was much slower, and only reached a maximum of 30 % after 24 h. Over a week, the API release from these three‐component beads rose to about 45 %.

At pH 4.0, in sodium acetate buffer, the drug release profiles were significantly different (Figure 4, bottom, Table S6). For the DBS‐COOH/alginate gel beads, the amount of drug released was less than at pH 7.4, only being about 45–50 %. In the DBS‐CONHNH_2_/alginate gel beads this value was only about 30 %, very similar to the calcium alginate beads. For the three‐component DBS‐COOH/DBS‐CONHNH_2_/alginate system, the drug release was now very much slower, and only reached a maximum of 7 % after 24 h.

We also considered drug release at pH 1.2 (Table S7, Figure S48), which is a good simulation of conditions in the stomach, relevant for oral delivery via the GI tract. In this case, we found that under these conditions, the DBS‐CONHNH_2_ gel network broke down, presumably as a result of protonation of the NH_2_ group. There is also the possibility of acetal hydrolysis leading to degradation of the gelator, however, DBS‐COOH was less affected, so we suggest this is a slower process. The release from gels containing DBS‐CONHNH_2_ was therefore much higher under these conditions than at pH 4, reaching 60 %+, presumably as a result of partial gel bead breakdown. For the DBS‐COOH/alginate beads, the release level was about 40 % after 24 h, slightly less than at pH 4.0. For calcium alginate beads, the release was only about 20 % at pH 1.2, once again slightly lower than at pH 4.0.

In order to interpret these results fully, it first needs to be considered that rosuvastatin is a weak acid with a p*K*_a_ of 4.6.^[21]^ Taking into consideration the Henderson‐Hasselbach equation, this means that at pH 7.4, >99 % of the drug is in its ionised form, which has the highest solubility in water and can therefore be more easily released from the gels. Conversely, at pH 4.0, the drug will be largely protonated, with lower solubility and release of the drug will therefore be more challenging. This inherent physicochemical property of rosuvastatin is reflected in the data, which indicates significantly greater API release for all gels at pH 7.4 than at pH 4.0. We consider the p*K*_a_ to be the primary factor controlling the release of the API in this case. However, on analysing the data in more detail, it is clear that the formulation of the gel bead also impacts significantly on the specifics of API release (see Discussion) and we consider this to be an important secondary factor controlling drug release in this case.

**Figure 5:**
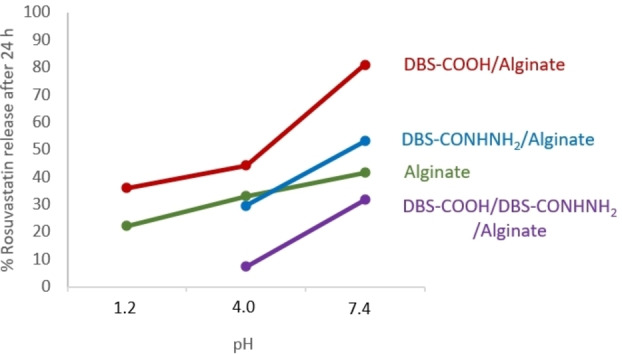
Summary of the effect of pH on % rosuvastatin release after 24 h from gel beads of different composition. Data for gel beads containing DBS‐CONHNH_2_ at pH 1.2 are omitted as the beads were not fully stable under these conditions.

Laid on top of this primary pH effect, there are also clear effects of gel bead composition on the release of rosuvastatin (Figure 5). It is evident for example, that the % release increases with pH, but that this effect is less significant for alginate alone than for those beads containing the LMWGs. This indicates that the gel bead composition modifies the pH effect on API release, particularly between pH 4.0 and 7.4. Furthermore, the precise choice of LMWG directly affects the amount of API released, with more release when using DBS‐COOH than DBS‐CONHNH_2_, which in turn has more release than the three‐component system containing both LMWGs. This clearly demonstrates that the chemical nature of the gel beads, as well as the p*K*_a_ of the API, are playing active roles in controlling API release.

In terms of comparing the performance of the different gel beads, it is firstly worth noting that the calcium alginate system on its own limits the release of rosuvastatin (this was also observed for release of the API from samples made in vials, so it is not just a function of the bead structure; Table S3, Figure S47). We suggest this limited API release is the result of interactions between rosuvastatin and the calcium alginate network, likely between the carboxylic acid of rosuvastatin and the divalent calcium ions in the PG. Interestingly, however, when DBS‐COOH is also present, there is significantly greater release of rosuvastatin, especially at pH 7.4 than at lower pH values. We suggest that as the pH rises, and the DBS‐COOH deprotonates causing the self‐assembled network to disassemble, the resulting LMWG carboxylate ion can itself interact with the calcium alginate PG network (this would also explain why not all of the DBS‐COOH is released into free solution from the gel in basic conditions, as described above and determined using NMR methods). This competitive effect of the DBS‐COOH would enable greater release of rosuvastatin from the gel. In this way, we suggest that the pH‐responsiveness of the DBS‐COOH gel beads triggers enhanced release of the API.

It is known that DBS‐CONHNH_2_ can specifically interact with acidic drugs at low pH values.^[10]^ A similar effect seems to be operational here, with just 30 % rosuvastatin release from the DBS‐CONHNH_2_/alginate system at pH 4.0, but 60 % release at pH 7.4. In this case, on raising the pH from 4.0 to 7.4, noncovalent interactions between the DBS‐CONHNH_2_ gel network and rosuvastatin are being switched off as the drug gets deprotonated. It is possible that the presence of the alginate network still slightly limits the release of the drug at the higher pH value by forming interactions with the API, this effect would be magnified by the core shell structure of these beads because the shell is based on calcium alginate.

For the three‐component system, drug release is low under both pH conditions, but especially so at pH 4.0. We suggest that the pH differences are primarily largely induced by the ionisation state of the rosuvastatin with greater release above its p*K*_a_ value. There will be a significant degree of interaction of the drug with DBS‐CONHNH_2_ below the p*K*_a_ value when the drug is protonated – this is potentially more marked than in the system that only had DBS‐CONHNH_2_/alginate, because the DBS‐COOH/DBS‐CONHNH_2_/alginate three‐component system is formed in the presence of an acid, which may further lower the internal pH of the gel bead, hence ensuring the rosuvastatin is fully protonated. Even at pH 7.4, drug release remains somewhat limited in this three‐component system. We note that in addition to the more densely packed network and the core‐shell structure, interactions between DBS‐CONHNH_2_ and DBS‐COOH (see above) mean that rosuvastatin can potentially also still interact with the calcium alginate network. As well as restricting the amount of API released, the release is also kinetically very significantly slower: after 2 h only about 17 % API has been released, rising to 30 % after 24 h and 45 % after 3 days. This suggests that this system only releases the drug slowly over time, possibly as a result of the denser, more interactive, multicomponent network.

## Conclusion

To conclude, we have fabricated pH‐responsive self‐assembled gel beads by combining the polymer gelator calcium alginate with the LMWGs DBS‐COOH and DBS‐CONHNH_2_, thus demonstrating for the first time that a range of LMWGs, assembled via different triggers, can be incorporated into gel beads by using an alginate‐based methodology. The polymer gelator enhances the thermal stability and the mechanical properties of the hybrid gels compared to the gels formed by the individual components. The rheological performance varies depending on the percentage of alginate incorporated, thereby giving gels with a range of stiffnesses. We demonstrated that the DBS‐COOH network could be selectively disassembled in response to pH, thus making these responsive gels potentially appealing for drug‐delivery applications.

**Figure 6:**
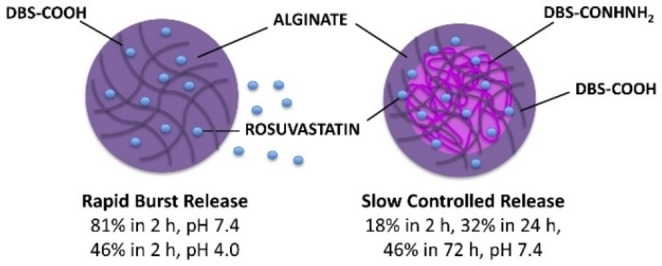
Summary of the different types of rosuvastatin release obtained from gel beads with different compositions.

In particular, we explored the release of the statin drug rosuvastatin calcium, a lipid‐lowering medication, in different pH buffers. The release of rosuvastatin calcium is pH‐dependent and also depends on the composition of the gel bead. Specifically, the presence of pH‐responsive DBS‐COOH enhances delivery, whereas interactive DBS‐CONHNH_2_ appears to limit drug release, especially at acidic pH values and in the three‐component system. By tuning the composition of the gel beads, we are able to either achieve rapid, almost complete release of the API with the DBS‐COOH/alginate beads, or alternatively much slower, more controlled release over a multi‐day timescale with the three‐component gel bead system (Figure 6).

Such different profiles of drug release might, in the future, be useful in different settings, with the former having potential for applications in bone regrowth or dentistry, which require rapid local release, but the latter being potentially useful for the development of slow systemic drug‐release applications for the treatment of hypercholesterolemia over the longer term. We therefore believe that our multicomponent gel beads could potentially be further developed as tuneable dosage systems for a variety of drugs, and could be a versatile platform for drug‐delivery applications. In this regard, it will be important to optimise drug loading into such gel beads – in the study here, we have focused on ensuring rosuvastatin is soluble at all times to facilitate analysis. As such, the total amounts of API released are somewhat below the therapeutic level. In future work, we intend to maximise the loading of the drug into the beads, even if in solid form. This should provide greater potential for the release of therapeutic levels of the drug over extended timescales. This will be an important next step in preclinical development. It will also be necessary to consider carefully how the delivery vehicle can best be administered. We note that, with approximately 3 mm diameter, the beads reported here are too large for most local/systemic applications, which would better make use of injection as a delivery mode. However, we have recently demonstrated the potential to develop injectable microgels from this type of system,^[9]^ and we therefore reason that statin‐loaded injectable microgel beads might be achievable. Such systems would be potentially transformative in injectable local and systemic delivery, and work towards this goal is currently a key target in our laboratory.

## Conflict of interest

The authors declare no conflict of interest.

## Supporting information

As a service to our authors and readers, this journal provides supporting information supplied by the authors. Such materials are peer reviewed and may be re‐organized for online delivery, but are not copy‐edited or typeset. Technical support issues arising from supporting information (other than missing files) should be addressed to the authors.

Supporting Information
