# Supplementary material for: Hybrid Self‐Assembled Gel Beads for Tuneable pH‐Controlled Rosuvastatin Delivery
Source: Chemistry. 2021 Aug 19;27(52):13203–10. doi: 10.1002/chem.202101405 (PMC8519141; doi:10.1002/chem.202101405)
Supplement: Supplementary file 1 — Supporting Information [file CHEM-27-13203-s001.pdf]

Chemistry–A European Journal

Supporting Information

Hybrid Self-Assembled Gel Beads for Tuneable pH-Controlled Rosuvastatin Delivery

Carmen C. Piras, Anna K. Patterson, and David K. Smith*

SUPPORTING INFORMATION

S1 General Experimental Methods

S2 Gel Preparation

S3 Synthesis and Characterisation of Gels in Vials

S4 NMR Assays

S5 Infrared (IR) Spectroscopy

S6 Optical Microscopy

S7 Transmission and Scanning Electron Microscopy (TEM and SEM)

S8 Thermal Stability Studies

S9 Rheology

S10 Drug Release Studies

S11 References

S1 General Experimental Methods

All compounds used in synthesis and analysis were purchased from standard commercial suppliers and used as received. The alginate employed in all the experiments was bought from Sigma Aldrich as sodium salt (2% viscosity). The synthesis of DBS-CONHNH$_2$ and DBS-COOH was performed in good yields applying previously reported methods.[1,2] ^{1}H NMR spectra were recorded using a Jeol 400 spectrometer (^{1}H 400 MHz). Samples were prepared in DMSO-d$_6$ and chemical shifts (δ) are reported in parts per million (ppm). IR spectra of xerogels were recorded on a PerkinElmer Spectrum Two FT-IR spectrometer. Optical microscopy images were obtained using a Zeiss Axiocam camera on a Zeiss stereo microscope. SEM images were taken using a JEOL JSM-7600F field emission SEM. TEM images were obtained on a FEI Tecnai 12 G^2 fitted with a CCD camera. Fibre sizes and gel bead diameters were measured using the *ImageJ* software. T_{gel} values were obtained using a high precision thermoregulated oil bath using the tube inversion method and were recorded in triplicate. Rheology was measured on a Malvern Instruments Kinexus Pro+ Rheometer fitted with a 20 mm parallel plate geometry.

S2 Gel Preparation

S2.1 DBS-COOH gels. DBS-COOH (0.3 or 0.4 % wt/vol) was suspended in water (1 mL) and dissolved by addition of a 0.5 M solution of NaOH (60 μL). The solution was then transferred into another sample vial containing GdL (1.0 % wt/vol) and left undisturbed overnight to allow gel formation.

S2.2 DBS-COOH/DBS-CONHNH$_2$ two-component gels. DBS-COOH (0.3 % wt/vol in 1 mL final total volume) was suspended in water (0.5 mL) and dissolved by addition of a 0.5 M solution of NaOH (60 μL). This solution was then added to a suspension of DBS-CONHNH$_2$ (0.3 % wt/vol in 1 mL final total volume) in water (0.5 mL). The suspension was sonicated to help the dispersion of the solid particles and subsequently heated until complete dissolution of the DBS-CONHNH$_2$. The hot solution was then transferred to another sample vial containing GdL (1.0 % wt/vol) and left undisturbed overnight to allow gel formation.

S2.3 Alginate gels. Alginate gels were prepared by adding a $CaCl_2$ solution (5.0% wt/vol – 1 mL) to an aqueous alginate solution (0.4-1.3% wt/vol). Gelation occurred immediately. The excess of $CaCl_2$ solution was then removed and the gels were washed with water multiple times.

S2.4 DBS-COOH/alginate two-component gels. DBS-COOH (0.3 % wt/vol in 1 mL final total volume) was suspended in water (0.5 mL) and dissolved by addition of a 0.5 M solution of NaOH (60 μL). An aqueous alginate solution (1.0% wt/vol - 0.5 mL) and GdL (1.0 % wt/vol) were then added. The amount of alginate and water was adjusted depending on the desired final concentration of the polymer in the different experiments. The sample was left undisturbed overnight to allow the formation of the DBS-COOH network. After 16 hours, a solution of $CaCl_2$ (5.0 % wt/vol – 1 mL) was then added on top of each gel to crosslink the alginate chains for 30 min. The excess of $CaCl_2$ solution was then removed and the gels were washed with water multiple times.

S2.5 DBS-CONHNH$_2$/DBS-COOH/alginate multicomponent gels. DBS-COOH (0.3 % wt/vol in 1 mL final total volume) was suspended in water (0.2 mL) and dissolved by addition of a 0.5 M solution of NaOH (60 μL). This solution was then added to a suspension of DBS-CONHNH$_2$ (0.3 % wt/vol in 1 mL final total volume) in water (0.24 mL). The suspension was sonicated to help the dispersion of the solid particles. An aqueous alginate solution (1.0% wt/vol - 0.5 mL) was then added. The amount of alginate and water was adjusted depending on the desired final concentration of the polymer in the different experiments. The sample was subsequently heated until complete dissolution of the DBS-CONHNH$_2$. The hot solution was then transferred to another sample vial containing GdL (1.0 % wt/vol, unless otherwise specified) and left undisturbed overnight to allow gel formation. After 16 hours, a solution of $CaCl_2$ (5.0 % wt/vol – 1 mL) was then added on top of each gel to crosslink the alginate chains for 30 min. The excess of $CaCl_2$ solution was then removed and the gels were washed with water multiple times.

S2.6 Alginate gel beads. Alginate gel beads were prepared by dropwise addition (20 μL/drop) of an aqueous alginate solution (0.8% wt/vol) to a $CaCl_2$ solution (5.0% wt/vol). The obtained beads were collected by filtration and washed with water multiple times.

S2.7 DBS-COOH/alginate gel beads. DBS-COOH (0.3 % wt/vol in 1 mL final total volume) was suspended in water (0.5 mL) and dissolved by addition of a 0.5 M solution of NaOH (60 μL). An

aqueous alginate solution (1.0% wt/vol - 0.5 mL) was subsequently added. The resulting solution was then added dropwise (20 µL/drop) to an acidic $CaCl_2$ solution (5.0% wt/vol - 25 mL, acidified with 250 µL of HCl 1M). To make sure that the DBS-COOH gelled completely, the obtained beads were left undisturbed in the $CaCl_2$ bath for 30 mins. After this time, they were collected by filtration and washed with water multiple times.

S2.8 DBS-COOH/DBS-$CONHNH_2$/alginate multicomponent gel beads. DBS-COOH (0.3 % wt/vol in 1 mL final total volume) was suspended in water (0.2 mL) and dissolved by addition of a 0.5 M solution of NaOH (60 µL). This solution was then added to a suspension of DBS-$CONHNH_2$ (0.3 % wt/vol in 1 mL final total volume) in water (0.24 mL). The suspension was sonicated to help the dispersion of the solid particles and an aqueous alginate solution (1.0% wt/vol - 0.5 mL) was then added. The amount of alginate and water was adjusted depending on the desired final concentration of the polymer in the different experiments. The sample was subsequently heated until complete dissolution of the DBS-$CONHNH_2$. The hot solution was then added dropwise (20 µL/drop) to an acidic $CaCl_2$ solution (5.0% wt/vol - 25 mL, acidified with 250 µL of HCl 1M). To make sure that the LMWGs completely, the obtained beads were left undisturbed in the $CaCl_2$ bath for 30 mins. After this time, they were collected by filtration and washed with water multiple times.

S3 Synthesis and Characterisation of Gels in Vials.

Alongside the studies of gel beads reported in the main paper, we also performed a study of bulk gels formed in vials – the characterisation studies are discussed here in detail. The drug release Section S10, alongside the results from the gel beads.

S3.1 Extended Interpenetrated Network Alginate/DBS-COOH Gels in Vials. Although alginic acid hydrogels can be obtained at acidic pH using GdL, to be sure that the alginate chains were fully cross-linked, we decided to combine the pH trigger of DBS-COOH with the Ca^{2+} ionic trigger. The DBS-COOH/alginate hybrid hydrogel was prepared using a stepwise approach by combining a basic aqueous solution of DBS-COOH (0.3% wt/vol) with sodium alginate (0.5% wt/vol) in the presence of GdL (1.0% wt/vol). Once the formation of the first gel network was completed, we ensured the

cross-linking of the alginate chains by addition of a $CaCl_2$ solution (5.0% wt/vol), which was added on top of the gel and left to diffuse through it.

We used microscopy to gain insight into the nanoscale fibrillar gel network. TEM and SEM analysis show a dense network of fibres with a diameter of 10-30 nm (Fig. S14 and S16). Due to the similarity of the different fibres it is not possible to distinguish between the two different components.

The supramolecular interactions between the DBS-COOH and the alginate network were investigated by IR spectroscopy. A clear shift of the O-H stretch of alginate from 3410 cm^{-1} to 3387 cm^{-1} is visible in the presence of the DBS-COOH network for the different hybrid gels (Fig. S7). The band for the C=O stretch of the DBS-COOH also shifted to lower frequencies in the presence of increasing alginate concentrations (Fig. S7). All these shifts are indicative of supramolecular interactions between the two gel networks.

We studied the macroscopic properties of the hybrid gels in terms of thermal stability and rheological performance and compared them to the properties of the gels obtained from the individual components. The thermal stability of the gels was evaluated by assessment of the sol-gel transition temperature (T_{gel}) by tube inversion method at increasing temperatures. The T_{gel} of the DBS-COOH hydrogel (78.5 °C) increases to > 100 °C for increased alginate loadings, showing that the presence of the polymer improves the gel thermal stability. Combination with alginate also improves the rheological performance of the DBS-COOH gels. Oscillatory rheology studies showed that the elastic modulus (G') of the DBS-COOH gels (0.4% wt/vol - G' = 360 Pa) progressively rises to 905 Pa, 2659 Pa, 5300 and 12000 Pa for increasing alginate concentrations (respectively 0.1, 0.3, 0.5 and 1.0% wt/vol, Section S9). This confirms that the presence of the polymer network increases the overall mechanical properties of the two-component gel due to the formation of supramolecular interactions between the two different self-assembled systems. The DBS-COOH gel elasticity, however, decreases in the presence of alginate, with shorter linear viscoelastic (LVE) regions for the two-component gels (Section S9). The crossover point (G' = G") for the DBS-COOH gel is at a 25.1% shear strain and it drops to 12.6%, 4.5%, 7.5% and 9.2% in the presence of higher alginate loadings (respectively 0.1, 0.3, 0.5 and 1.0% wt/vol, Section S8). The

DBS-COOH/alginate two-component gels are therefore stiffer, but more brittle than the gel formed by the LMWG alone.

S3.2 Extended Interpenetrating Network DBS-COOH/DBS-CONHNH$_2$/alginate Gels in Vials. To further modify the performance of the DBS-COOH/alginate two-component gels, whilst keeping the pH responsiveness, we decided to add the LMWG DBS-CONHNH$_2$ to our formulation. DBS-CONHNH$_2$ forms thermally triggered hydrogels at low concentrations (0.3% wt/vol) by heat-cool cycle. DBS-COOH/DBS-CONHNH$_2$ gels were previously reported by us and were obtained by combining a basic aqueous solution of DBS-COOH (0.2 or 0.3% wt/vol) with DBS-CONHNH$_2$ (0.2 or 0.3% wt/vol).[3] The mixture was heated until complete dissolution of the DBS-CONHNH$_2$, then transferred to another sample vial containing GdL (0.8 or 1.0 % wt/vol) and left undisturbed overnight to allow gel formation. In summary, we studied the properties of DBS-COOH/DBS-CONHNH$_2$ gels and demonstrated that these gels were stiffer than those formed by the individual components. Moreover, the self-assembly of the DBS-COOH network could be "switched on" and "off" by pH variations within the hybrid gel, with minimal effects on the self-assembly of the DBS-CONHNH$_2$ network (see detailed discussion below).

DBS-COOH/DBS-CONHNH$_2$/alginate gels were prepared by applying the procedure described above in the presence of alginate (0.5% wt/vol) using a 0.3% wt/vol concentration of each LMWG and 1.0% GdL. Once the two LMWGs networks were formed, a $CaCl_2$ solution (5.0% wt/vol) was added on top of the gel and let diffuse through it, to cross-link the PG.

The morphology of the multicomponent gel fibers was analysed by TEM and SEM. Both techniques showed a complex network of densely packed fibers with a diameter of 9-33 nm (Fig S15 and S17).

The macroscopic properties of the DBS-COOH/DBS-CONHNH$_2$/alginate gels were studied in terms of thermal stability and rheology. The sol-gel transition temperature (T_{gel}) was assessed by the tube inversion method as described above for the DBS-COOH/alginate gels. The combination of the three gelators prepared using different alginate concentrations (0.1-1.0% wt/vol) allowed obtaining thermally stable gels with T_{gel} values higher than 100 °C.

The mechanical properties of the DBS-COOH/DBS-CONHNH$_2$/alginate gels were studied by oscillatory rheology. The DBS-COOH/DBS-CONHNH$_2$ gel prepared using a 0.3% wt/vol concentration of the two LMWGs and a 1.0% wt/vol concentration of GdL showed an elastic modulus of 5410 Pa (Section S8). In the presence of increasing alginate concentrations (0.1, 0.3, 0.5 and 1.0 % wt/vol), the G' value increased to 6950, 15200, 19000 and 37300 Pa respectively (Section S8). These gels were stiffer than the DBS-COOH/DBS-CONHNH$_2$ gel, but showed shorter LVE regions and were therefore less elastic than the gels prepared without alginate, as was also observed for the DBS-COOH/alginate gels.

Finally, we wanted to demonstrate that we could selectively 'switch on and off' the DBS-COOH network within the multicomponent gel without affecting the gel mechanical properties after the DBS-COOH network was re-formed. To perform this test, we prepared the DBS-COOH/DBS-CONHNH$_2$/alginate gel using a 1.0% wt/vol concentration of alginate, which displayed an elastic modulus of 37300 Pa. We then selectively destroyed the DBS-COOH network by increasing the pH by addition of a small amount of NaOH (0.5M – 60 μL). The gel rheological performance was evaluated again after 24 hours and, although the gel kept its integrity, the G' dropped to 28300 Pa as a result of the disruption of the DBS-COOH network (Section S8). The self-assembly of DBS-COOH was then re-induced by addition of GdL (1.0% wt/vol). The elastic modulus of the multicomponent gel increased again to 34500 Pa as a result of the reformation of the DBS-COOH network (Section S8). This simple experiment shows how it is possible to control the gel stiffness and fibre packing by exploiting the gel pH responsiveness. This property makes these hybrid materials very interesting for drug delivery applications.

S4 NMR Assays

^{1}H NMR was employed to confirm that the LMWGs incorporated into the DBS-COOH/alginate and DBS-CONHNH$_2$/DBS-COOH/alginate gel beads were in self-assembled state and to quantify the exact amount of LMWGs loaded into each gel bead.

S4.1 NMR verification of the self-assembled state of DBS-COOH incorporated into the DBS-COOH/alginate gel beads. DBS-COOH (0.3 % wt/vol in 1 mL final total volume) was suspended in D_2O (0.5 mL) and dissolved by addition of a 0.5 M solution of NaOD (60 μL). An alginate solution in

D_2O (1.0% wt/vol - 0.5 mL) was subsequently added. The resulting solution was then added dropwise (20 μL/drop) to an acidic $CaCl_2$ solution in D_2O (5.0% wt/vol - 5 mL, acidified with 50 μL of DCl 1M). Ten gel beads were isolated and transferred into a NMR tube. D_2O (0.5 mL) and anhydrous MeCN (3.0 μL) were then added. The ^1H NMR spectrum was recorded and the amount of mobile components was calculated by comparison of the integrals of relevant peaks (DBS-COOH aromatic peaks δ = 7.55 and 7.95) to that of acetonitrile (δ = 2.09 ppm).

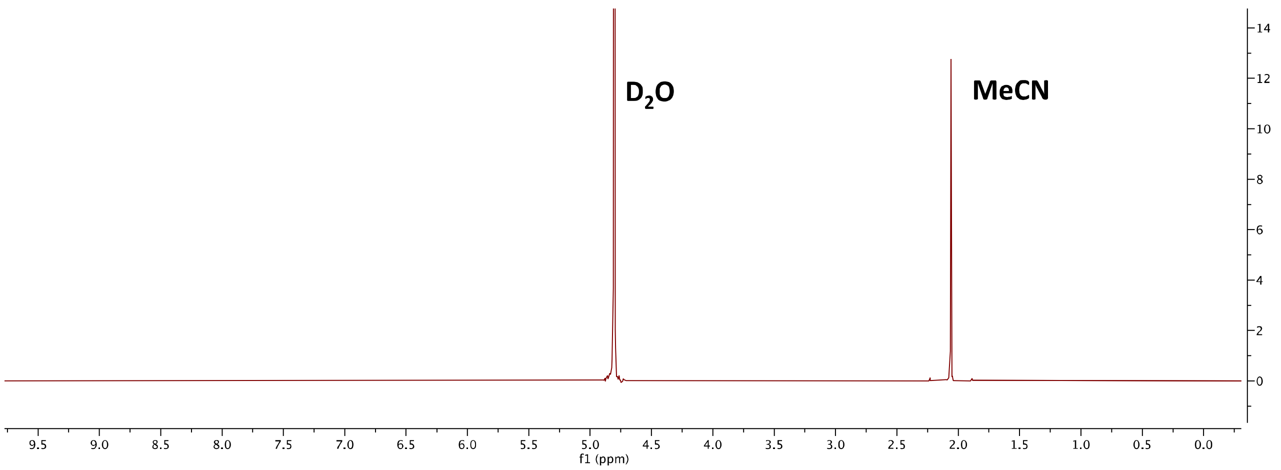

Figure S1. ^1H NMR of ten DBS-COOH/alginate gel beads in D_2O.

S4.2 NMR quantification of DBS-COOH incorporated in each DBS-COOH/alginate gel bead. The gel beads used for this experiment were prepared by combining DBS-COOH (0.3 % wt/vol) and alginate (0.5 % wt/vol) in water (1 mL) as described in Section S2.7. Ten gel beads were isolated and dried under high vacuum. The resulting solid was dissolved in DMSO-d_6 (0.7 mL), and acetonitrile (3.0 μL) was added as an internal standard. To make sure that all the DBS-COOH was dissolved, the sample was ground and then sonicated for 30 min. The ^1H NMR spectrum was recorded and the concentration of the LMWG calculated by comparison of the integrals of relevant peaks (DBS-COOH aromatic peaks δ = 7.55 and 7.95 ppm) to that of acetonitrile (δ = 2.09 ppm). To ensure reproducibility, this experiment was repeated on two batches of gel beads. It is noted that due to the low solubility of alginate in DMSO-d_6, the alginate peaks were not visible.

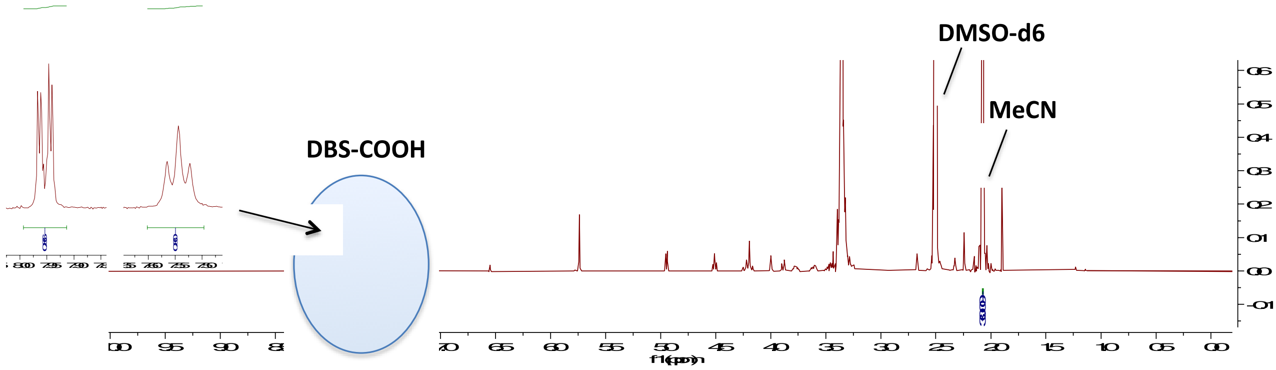

Figure S2. ^{1}H NMR of DBS-COOH incorporated into ten DBS-COOH/alginate gel beads.

S4.3 NMR quantification of mobile DBS-COOH in DBS-COOH/alginate gel beads in which the DBS-COOH network was disrupted. Ten gel beads were prepared in D_2O as described in S.3.4 and transferred into a NMR tube. D_2O (0.5 mL), NaOD (60 μL) and anhydrous MeCN (3.0 μL) were then added. The sample was left undisturbed overnight and the ^{1}H NMR spectrum was then recorded. The percentage of mobile components was calculated by comparison of the integrals of relevant peaks (DBS-COOH aromatic peaks δ = 7.55 and 7.95 ppm) to that of acetonitrile (δ = 2.09 ppm).

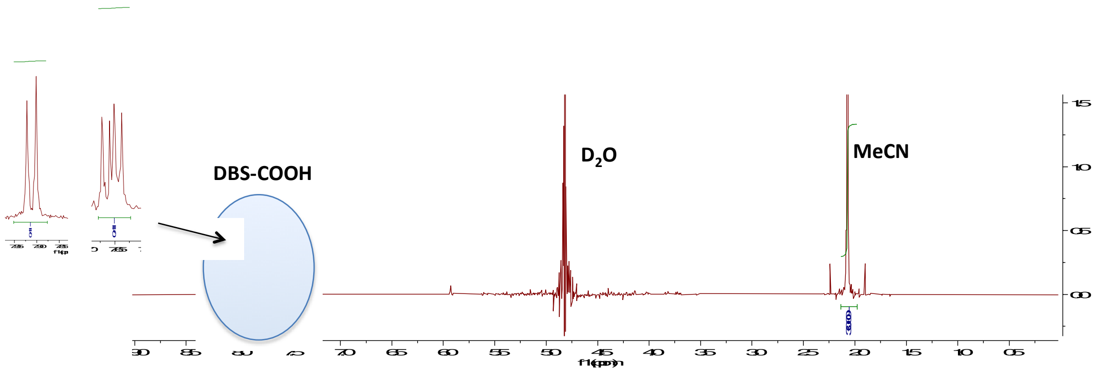

Figure S3. ^{1}H NMR of mobile DBS-COOH after disruption of the DBS-COOH network in ten DBS-COOH/alginate gel beads.

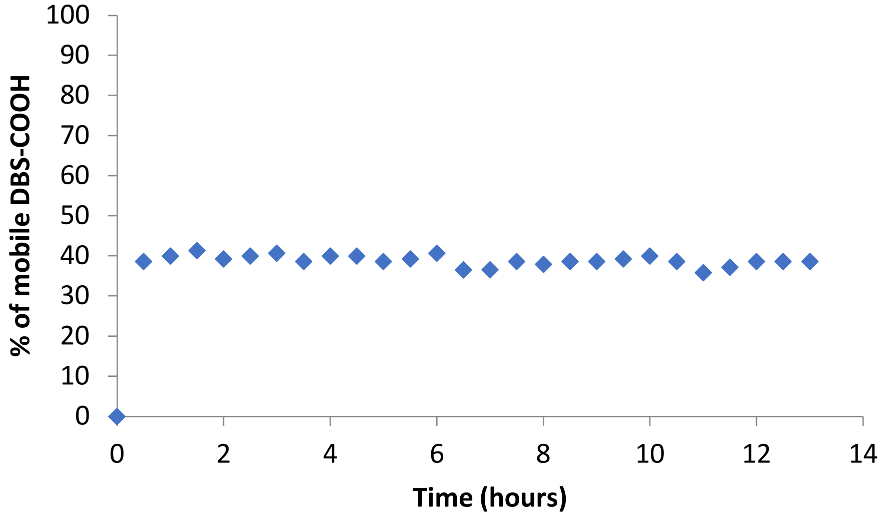

Figure S4. Percentage of mobile DBS-COOH over time after addition of NaOD to ten DBS-COOH/alginate gel beads.

S4.4 NMR verification of the self-assembled state of the LMWGs incorporated into the DBS-COOH/DBS-CONHNH₂/alginate gel beads. DBS-COOH (0.3 % wt/vol in 1 mL final total volume) was suspended in D_2O (0.2 mL) and dissolved by addition of a 0.5 M solution of NaOD (60 μL). This solution was then added to a suspension of DBS-$CONHNH_2$ (0.3 % wt/vol in 1 mL final total volume) in D_2O (0.24 mL). The suspension was sonicated to help the dispersion of the solid particles. An alginate solution in D_2O (1.0% wt/vol - 0.5 mL) was then added. The sample was subsequently heated until complete dissolution of the DBS-$CONHNH_2$. The hot solution was then then added dropwise (20 μL/drop) to an acidic $CaCl_2$ solution in D_2O (5.0% wt/vol - 5 mL, acidified with 50 μL of DCl 1M). Ten gel beads were isolated and transferred into a NMR tube. D_2O (0.5 mL) and anhydrous MeCN (3.0 μL) were then added. The ^1H NMR spectrum was recorded and the amount of mobile components was calculated by comparison of the integrals of relevant peaks (DBS-COOH aromatic peak δ = 7.95 and DBS-$CONHNH_2$ aromatic peak δ = 7.82) to that of acetonitrile (δ = 2.09 ppm).

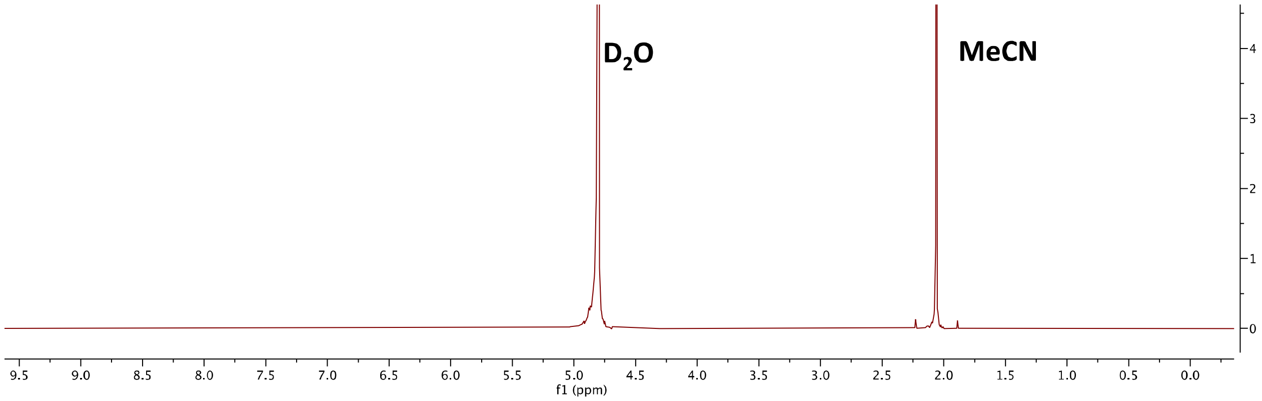

Figure S5. ^1H NMR of ten DBS-COOH/DBS-CONHNH$_2$/alginate beads in D_2O.

S4.5 NMR quantification of the LMWGs incorporated into the DBS-COOH/DBS-CONHNH$_2$/alginate gel beads. The gel beads used for this experiment were prepared by combining DBS-COOH (0.3 % wt/vol), DBS-CONHNH$_2$ (0.3 % wt/vol) and alginate (0.5 % wt/vol) in water (1 mL) as described in Section S2.8. Ten gel beads were isolated and dried under high vacuum. The resulting solid was dissolved in DMSO-d$_6$ (0.7 mL), and acetonitrile (3.0 µL) was added as an internal standard. To make sure that all the DBS-COOH was dissolved, the sample was ground and then sonicated for 30 min. The ^1H NMR spectrum was recorded and the concentration of the LMWG calculated by comparison of the integrals of relevant peaks (DBS- COOH aromatic peak δ = 7.95 and DBS-CONHNH$_2$ aromatic peak δ = 7.82) to that of acetonitrile (δ = 2.09 ppm). To ensure reproducibility, this experiment was repeated on two batches of gel beads. It is noted that due to the low solubility of alginate in DMSO-d$_6$, the alginate peaks were not visible.

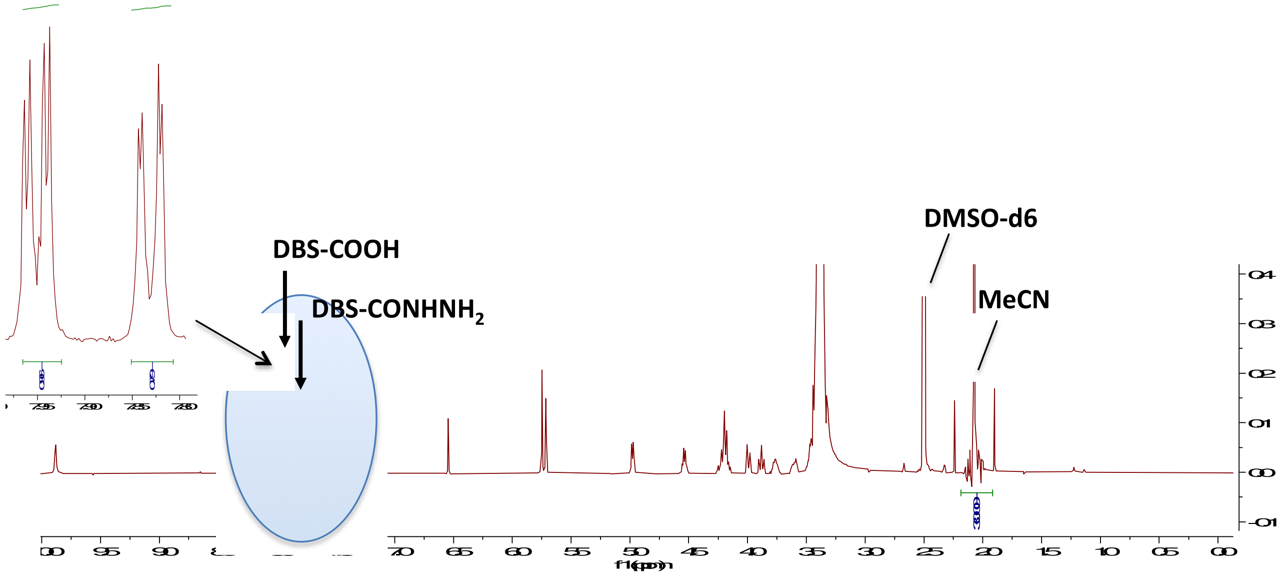

Figure S6. ^1H NMR of DBS-COOH and DBS-CONHNH$_2$ incorporated into ten DBS-COOH/DBS-CONHNH$_2$/alginate gel beads.

S4.6 NMR quantification of mobile LMWGs in DBS-COOH/DBS-CONHNH$_2$/alginate gel beads in which the DBS-COOH network was disrupted. Ten gel beads were prepared in D_2O as described in S.3.4 and transferred into a NMR tube. D_2O (0.5 mL), NaOD (60 µL) and anhydrous MeCN (3.0 µL) were then added. The sample was left undisturbed overnight and the ^{1}H NMR spectrum was then recorded. The percentage of mobile components was calculated by comparison of the integrals of relevant peaks (DBS-COOH aromatic peak δ = 7.95 and DBS-CONHNH$_2$ aromatic peak δ = 7.82) to that of acetonitrile (δ = 2.09 ppm).

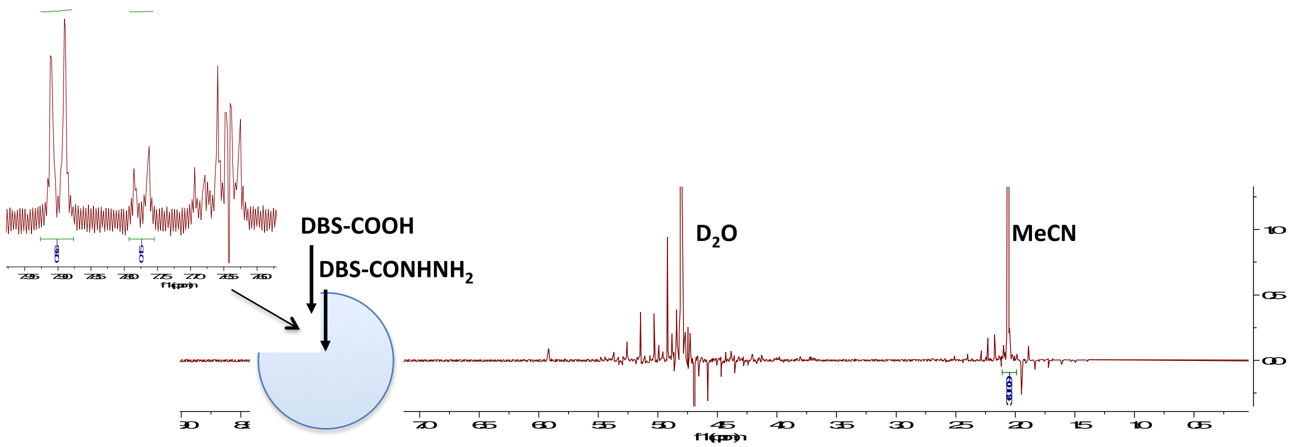

Figure S7. ^{1}H NMR of mobile DBS-COOH and DBS-CONHNH$_2$ after disruption of the DBS-COOH network in ten DBS-COOH/DBS-CONHNH$_2$/alginate gel beads.

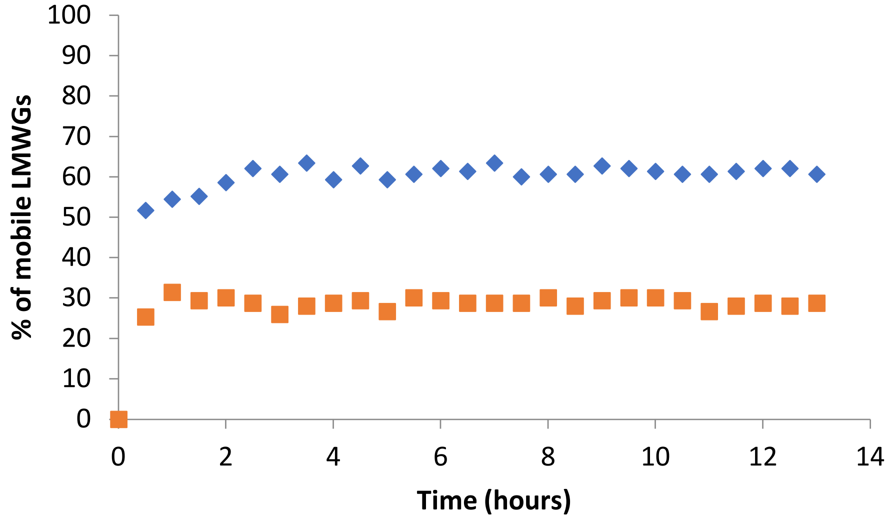

Figure S8. Percentage of mobile DBS-COOH (blue dots) and DBS-CONHNH$_2$ (orange dots) over time after addition of NaOD to ten DBS-COOH/DBS-CONHNH$_2$/alginate gel beads.

S5 Infrared (IR) Spectroscopy

Xerogel samples for infrared were prepared by removing the solvent from the gels under high vacuum. A small amount of the resulting powder was placed into the infrared spectrophotometer and the spectra recorded in the range of 450-4000 cm^{-1}.

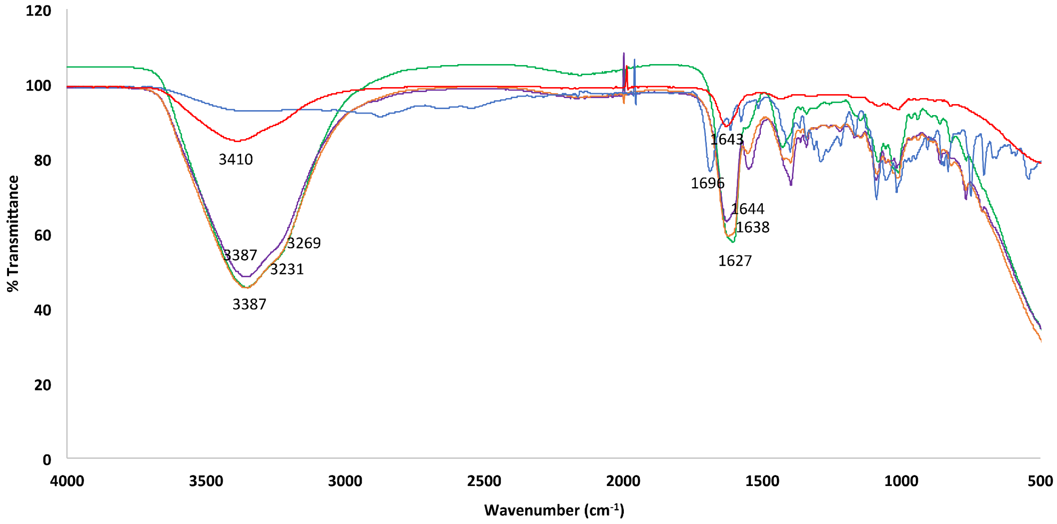

Figure S9. IR spectra of xerogels obtained from DBS-COOH gel (0.4% wt/vol, blue line), alginate gel (0.8% wt/vol, red line) and DBS-COOH/alginate gel containing 0.3% wt/vol of DBS-COOH and 0.3% wt/vol alginate (purple line), 0.5% wt/vol alginate (orange line) and 1.0% wt/vol alginate (green line).

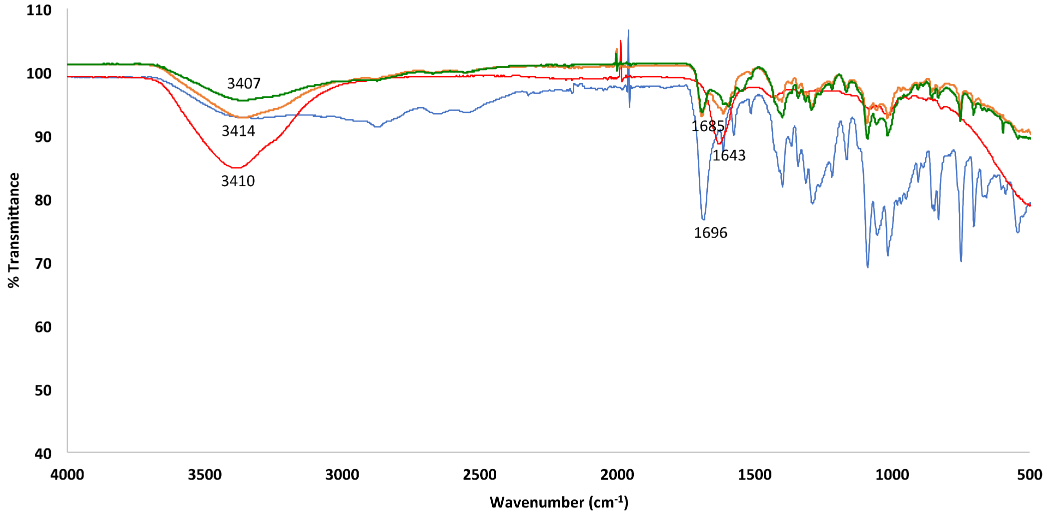

Figure S10. IR spectra of xerogels obtained from DBS-COOH gel (0.4% wt/vol, blue line), alginate gel beads (0.8% wt/vol, red line) and DBS-COOH/alginate gel beads containing 0.3% wt/vol of DBS-COOH and 0.5% wt/vol alginate (orange line) or 1.0% wt/vol alginate (green line).

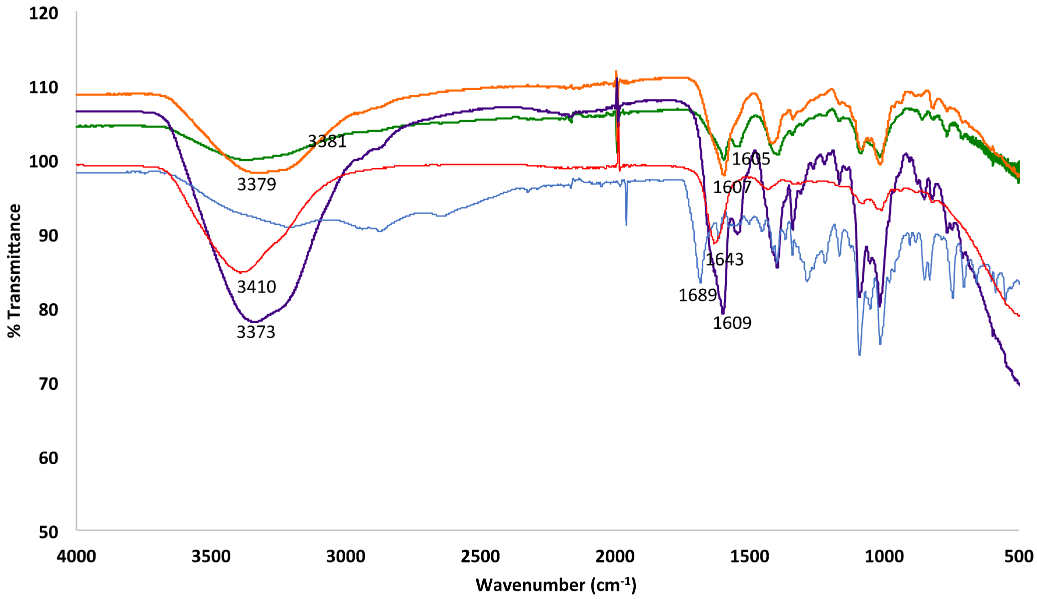

Figure S11. IR spectra of xerogels obtained from DBS-COOH/DBS-CONHNH$_2$ gel (0.3% wt/vol each LMWG, blue line), alginate gel (0.8% wt/vol, red line) and DBS-COOH/DBS-CONHNH$_2$/alginate gel containing 0.3% wt/vol of each LMWG and 0.3% wt/vol alginate (purple line), 0.5% wt/vol alginate (orange line) and 1.0% wt/vol alginate (green line).

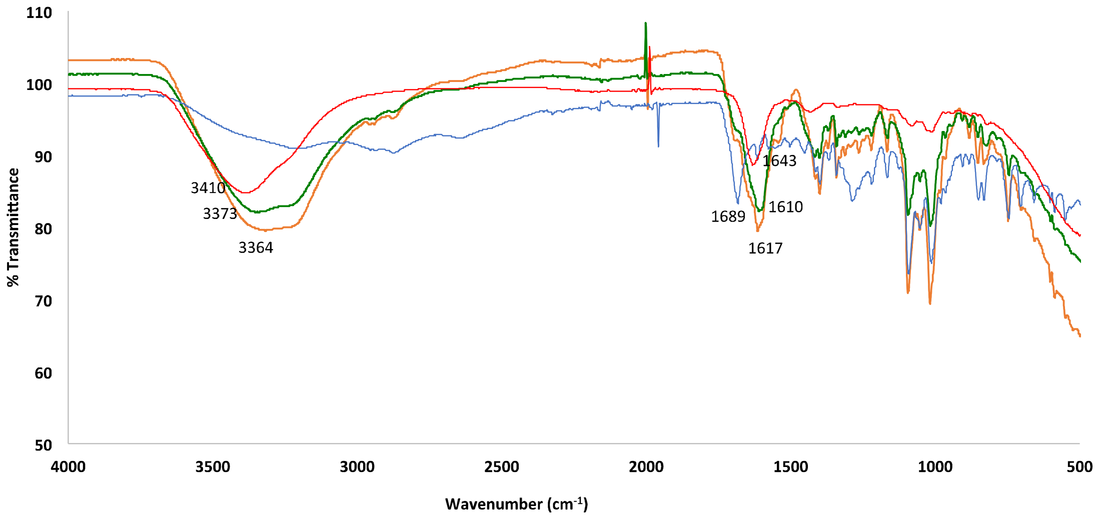

Figure S12. IR spectra of xerogels obtained from DBS-COOH/DBS-CONHNH$_2$ gel (0.3% wt/vol each LMWG, blue line), alginate gel beads (0.8% wt/vol, red line) and DBS-COOH/DBS-CONHNH$_2$/alginate gel beads containing 0.3% wt/vol of each LMWG and and 0.5% wt/vol alginate (orange line) or 1.0% wt/vol alginate (green line).

S6 Optical Microscopy

Optical microscopy images were collected on a Zeiss stereo microscope. The gel beads were either analysed as such or pre-embedded into resin. These last samples were dehydrated through an ethanol series, then embedded in LR white resin. Sections were 1 μm thick. Once the section was dried on the slide, it was stained with Toluidine Blue (0.6% with 0.3% Na_2CO_3). All the gel beads were prepared using 20 μL volumes (unless otherwise specified) using 0.3% wt/vol of DBS-$CONHNH_2$, 0.3% wt/vol of DBS-COOH and 0.5% wt/vol of alginate.

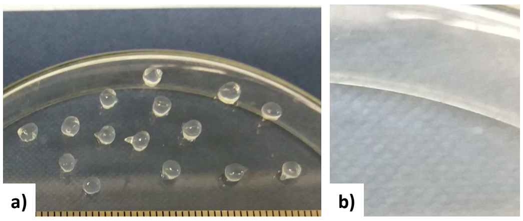

Figure S13. DBS-COOH/alginate gel beads prepared by addition of (a) 20 μL and (b) 5 μL volumes into a $CaCl_2$ solution (5%). A ruler was included to show the diameters.

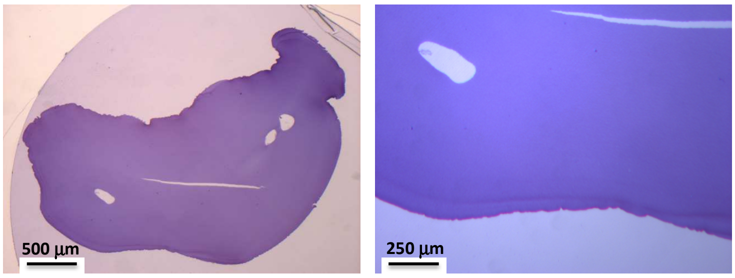

Figure S14. Cross-section of DBS-COOH/alginate two-component gel beads embedded in resin, coloured with toluidine blue. Scale bars: 500 μm (left) and 250 μm (right).

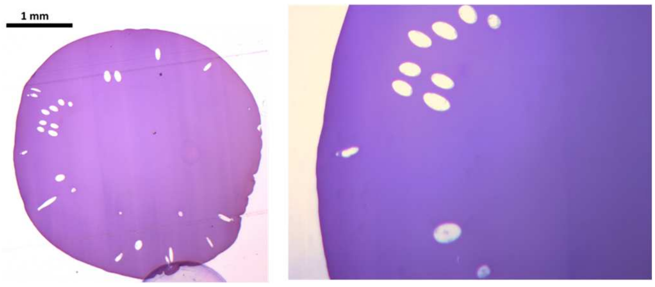

Figure S15. Cross-section of alginate gel beads embedded in resin, coloured with toluidine blue. Scale bar: 1 mm (left).

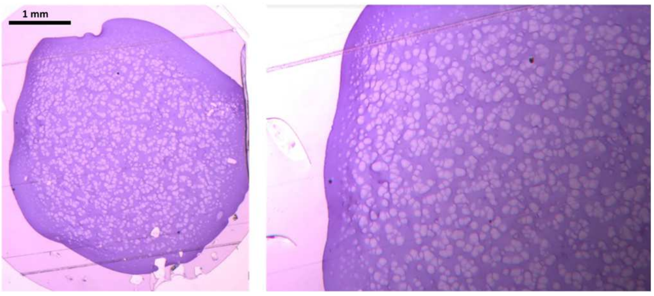

Figure S16. Cross-section of DBS-CONHNH$_2$/alginate two-component gel beads embedded in resin, coloured with toluidine blue. Scale bar: 1 mm (left).

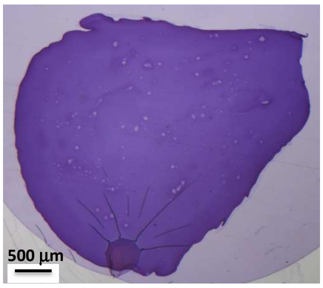

Figure S17. Cross-section of DBS-COOH/DBS-CONHNH$_2$/alginate gel bead embedded in resin and coloured using toluidine blue. Scale bar: 500 μm.

S7 Transmission and Scanning Electron Microscopy (TEM and SEM)

S7.1 Preparation of samples for TEM. Samples for TEM were obtained by placing a small amount of each sample on a copper grid. The excess of sample was removed with filter paper and allowed to set for 5 min. A negative stain (1% uranyl acetate) was then added and the samples were left to rest for 30 min before taking the images.

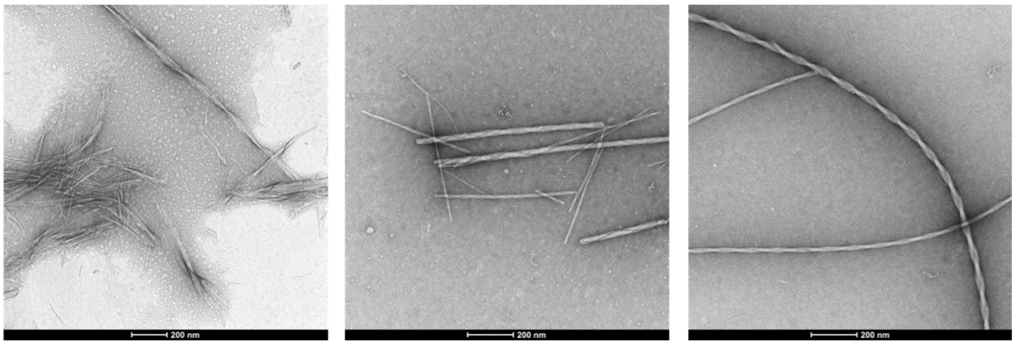

Figure S18. TEM images of DBS-COOH gel (left) and DBS-COOH/alginate two-component gel (centre and right). Scale bars: 200 nm.

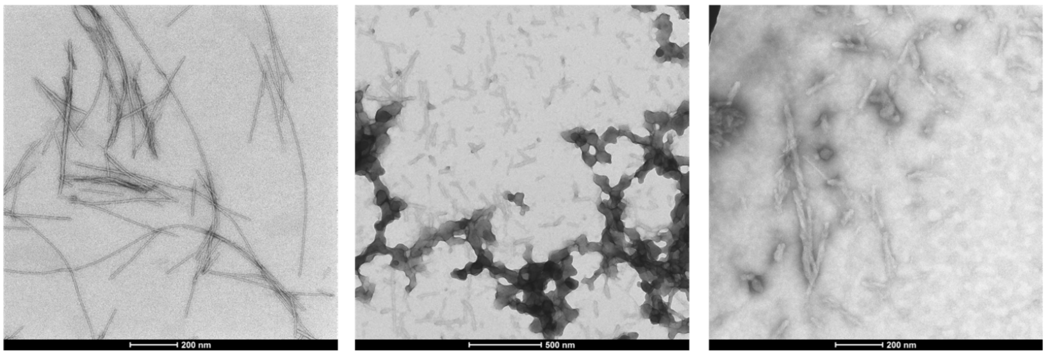

Figure S19. TEM images of DBS-COOH/DBS-CONHNH$_2$ gel (left) and DBS-COOH/DBS-CONHNH$_2$/alginate gel (centre and right). Scale bars from left to right: 100 nm, 5 and 1 μm.

S7.2 Preparation of samples for SEM. Samples for SEM were obtained by freeze-drying the gels on copper shim pieces. The freeze-dried samples were then mounted on stubs and the images recorded. Alginate and hybrid beads were critical point dried (acetone and liquid CO_2) and mounted on stubs either whole, or halved using a razor blade. Mounted samples were sputter coated with Au/Pd.

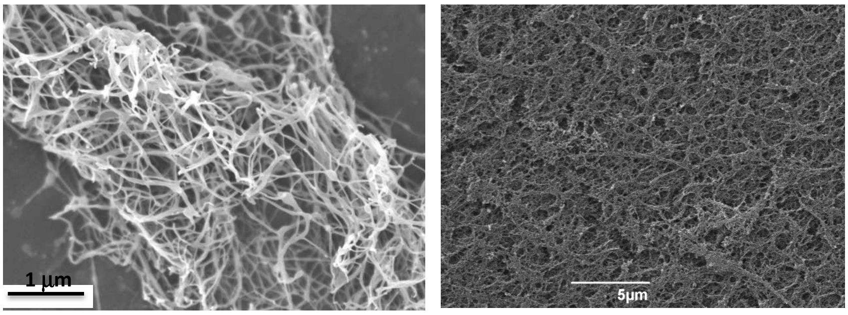

Figure S20. SEM images of DBS-COOH gel (left) and DBS-COOH/alginate gel (right). Scale bars: 1 μm (left) and 5 μm (right).

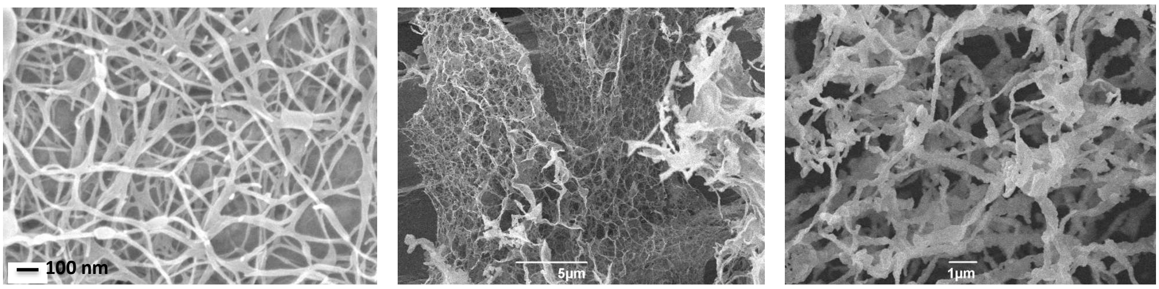

Figure S21. SEM images of DBS-COOH/DBS-CONHNH$_2$ gel (left) and DBS-COOH/DBS-CONHNH$_2$/alginate gel (centre and right). Scale bars from left to right: 100 nm, 5 and 1 μm.

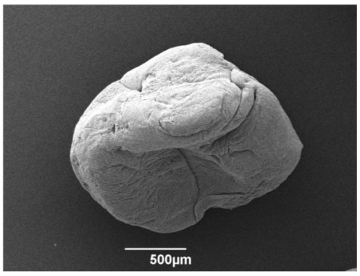

Figure S22. SEM images of DBS-COOH/alginate two-component gel bead. Scale bar: 500 µm.

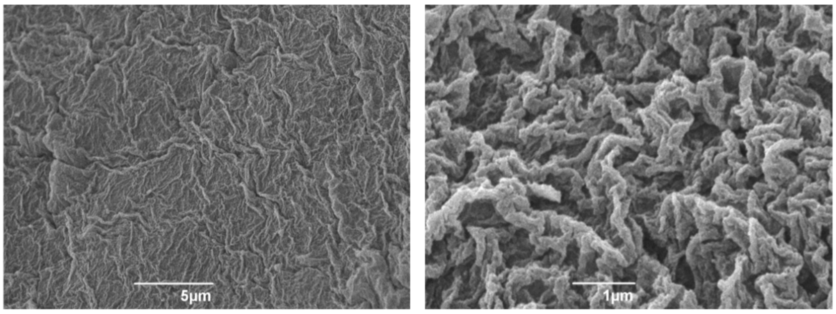

Figure S23. SEM images of DBS-COOH/alginate two-component gel bead surface. Scale bars: 5 µm (left) and 1 µm (right).

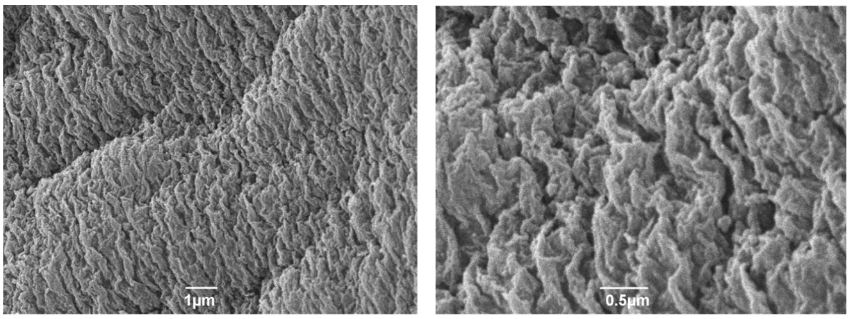

Figure S24. SEM images of DBS-COOH/DBS-CONHNH$_2$/alginate gel bead surface. Scale bars: 1 µm (left) and 0.5 µm (right).

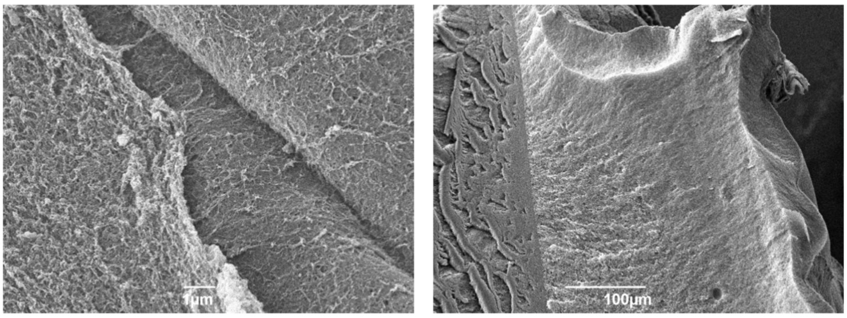

Figure S25. SEM images of DBS-COOH/alginate (left) and DBS-COOH/DBS-CONHNH$_2$/alginate gel bead cross-section (right). Scale bars: 200 µm (left) and 1 µm (centre and right).

S8 Thermal Stability Studies

All the gels for T_{gel} determination were prepared as described in Section S2 in 2 mL vials (diameter: 1 cm, height: 4 cm). All the gels were placed in a high precision thermoregulated oil bath with an initial temperature of 25°C. The temperature was increased by 1°C/ min until 100°C. Every minute the gels were checked by tube inversion method and T_{gel} was considered as the temperature at which the gel began to run down the sides of the vial. These experiments were performed in triplicate to ensure reproducibility and the average is reported. Errors are estimated at ±2°C.

Table S1. T_{gel} values of gels formed by individual gelators, the DBS-COOH/alginate gel and DBS-COOH/DBS-CONHNH$_2$/alginate multicomponent gel.

GEL (1 mL total volume)	Loading of DBS-CONHNH$_2$	Loading of DBS-COOH	Loading of Alginate	T_{gel}
DBS-COOH	-	0.4%	-	78.5 °C
DBS-CONHNH$_2$	0.4%	-	-	86 °C
Alginate	-	-	0.4%	>100 °C
Alginate	-	-	0.6%	>100 °C
Alginate	-	-	0.8%	>100 °C
Alginate	-	-	1.3%	>100 °C
DBS-COOH/Alginate	-	0.3%	0.1%	>100 °C
DBS-COOH/Alginate	-	0.3%	0.3%	>100 °C
DBS-COOH/Alginate	-	0.3%	0.5%	>100 °C
DBS-COOH/Alginate	-	0.3%	1.0%	>100 °C
DBS-COOH/DBS-CONHNH$_2$	0.3%	0.3%	-	>100 °C
Multicomponent gel	0.3%	0.3%	0.1%	>100 °C
Multicomponent gel	0.3%	0.3%	0.3%	>100 °C
Multicomponent gel	0.3%	0.3%	0.5%	>100 °C
Multicomponent gel	0.3%	0.3%	1.0%	>100 °C
Multicomponent gel	0.3%	0.3%	1.0%	>100 °C
Multicomponent gel	0.3%	0.3%	1.0%	>100 °C

S9 Rheology

Gel samples for rheology were prepared as described in Section S2 using bottomless vials as templates to obtain the intended gel dimensions. The measurements were carried out at 25°C using a 20 mm parallel plate and a gap of 2 mm. To avoid solvent evaporation and keep the

sample hydrated, a solvent trap was used, and the internal atmosphere was kept saturated. Amplitude sweep experiments were performed in the range of 0.05-100% strain at a 1 Hz frequency to identify the linear viscoelastic region. Frequency sweep experiments were performed between 0.1 and 100 Hz using a shear strain of 0.25%. The measurements were repeated three times to ensure reproducibility and the average data are shown.

Table S2. Rheological data as determined using oscillatory rheometry with a parallel plate geometry, for DBS-COOH gels, DBS-COOH/DBS-CONHNH$_2$ gels, calcium alginate gels, and multicomponent gels formed by the combination of the three individual gelators. Loadings are given in wt/vol, and the G'/G" crossover points refer to the % shear strain at which G"=G'.

Gel	Loading of DBS-CONHNH$_2$	Loading of DBS-COOH	Loading of Alginate	Total Loading	API Loading	G' (Pa)	G'/G" Crossover
DBS-COOH	-	0.4%	-	0.4%	-	360	25.1%
Alginate	-	-	0.4%	0.4%	-	490	6.5%
Alginate	-	-	0.6%	0.6%	-	1420	19.9%
Alginate	-	-	0.8%	0.8%	-	2500	2.3%
Alginate			0.8%	0.8%	Yes	1390	2.5%
Alginate	-	-	1.3%	1.3%	-	17100	8.5%
DBS-COOH/Alginate	-	0.3%	0.1%	0.4%	-	905	12.6%
DBS-COOH/Alginate	-	0.3%	0.3%	0.6%	-	2659	4.5%
DBS-COOH/Alginate	-	0.3%	0.5%	0.8%	-	5300	7.5%
DBS-COOH/Alginate		0.3%	0.5%	0.8%	Yes	3870	3.5%
DBS-COOH/Alginate	-	0.3%	1.0%	1.3%	-	12000	9.2%
DBS-CONHNH$_2$/Alginate	0.3%		0.5%	0.8%	-	8260	6.9%
DBS-CONHNH$_2$/Alginate	0.3%		0.5%	0.8%	Yes	9420	2.7%
DBS-COOH/DBS-CONHNH$_2$	0.3%	0.3%	-	0.6%	-	5410	12.7%
Three-component gel	0.3%	0.3%	0.1%	0.7%	-	6950	8.5%
Three-component gel	0.3%	0.3%	0.3%	0.9%	-	15200	4.3%
Three-component gel	0.3%	0.3%	0.5%	1.1%	-	19000	7.8%
Three-component gel	0.3%	0.3%	0.5%	1.1%	Yes	17700	3.5%
Three-component gel	0.3%	0.3%	1.0%	1.6%	-	37300	3.4%
Three-component gel: DBS-COOH disrupted	0.3%	0.3%	1.0%	1.6%	-	28300	8.6%
Three-component gel: DBS-COOH disrupted & re-formed	0.3%	0.3%	1.0%	1.6%	-	34500	6.5%

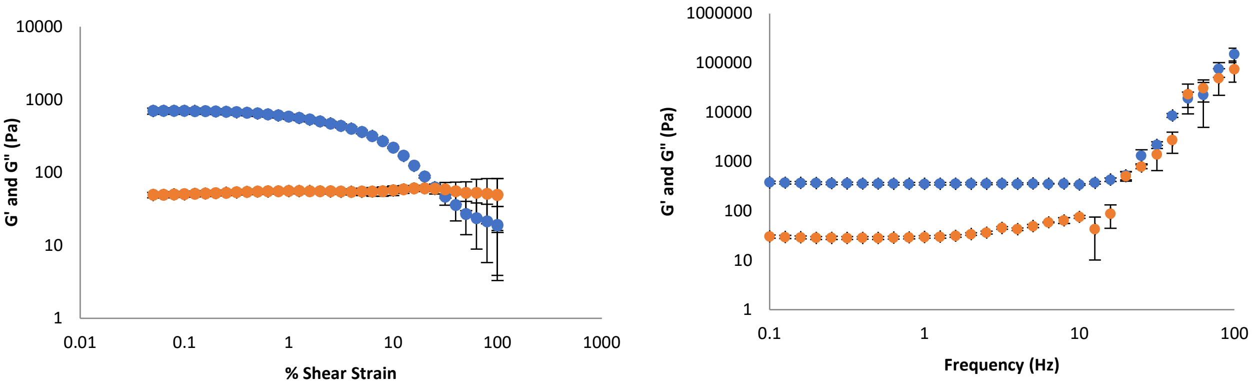

Figure S26. Elastic (G’, blue circles) and viscous (G”, orange circles) moduli of DBS-COOH hydrogel (0.4% wt/vol) with increasing shear strain (left) and frequency (right).

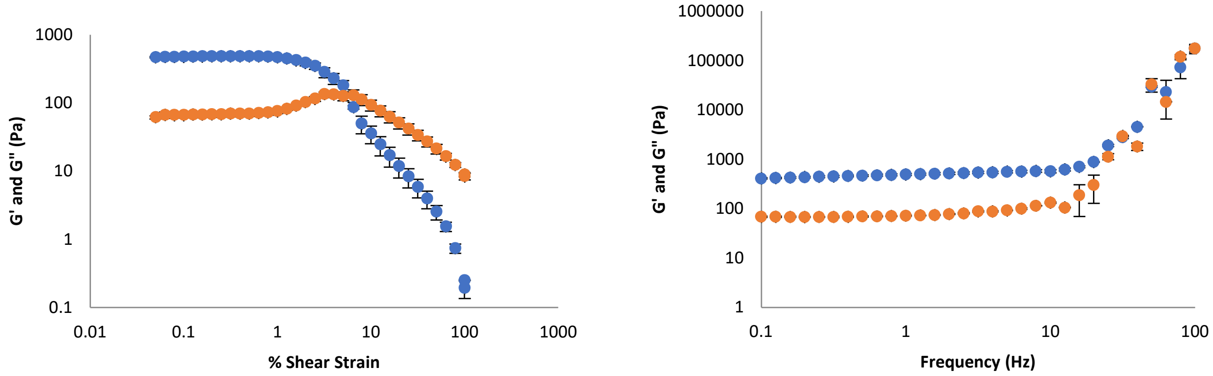

Figure S27. Elastic (G’, blue circles) and viscous (G”, orange circles) moduli of alginate hydrogel (0.4% wt/vol) with increasing shear strain (left) and frequency (right).

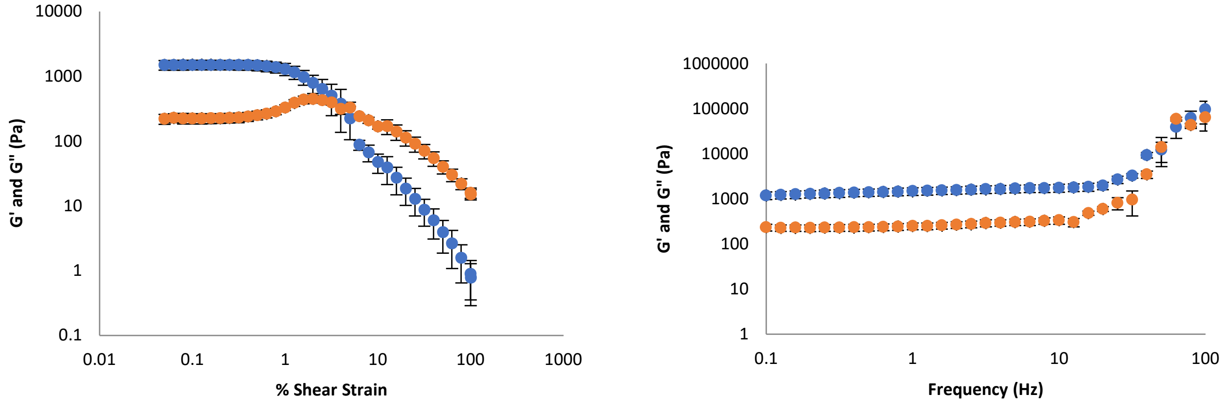

Figure S28. Elastic (G’, blue circles) and viscous (G”, orange circles) moduli of alginate hydrogel (0.6% wt/vol) with increasing shear strain (left) and frequency (right).

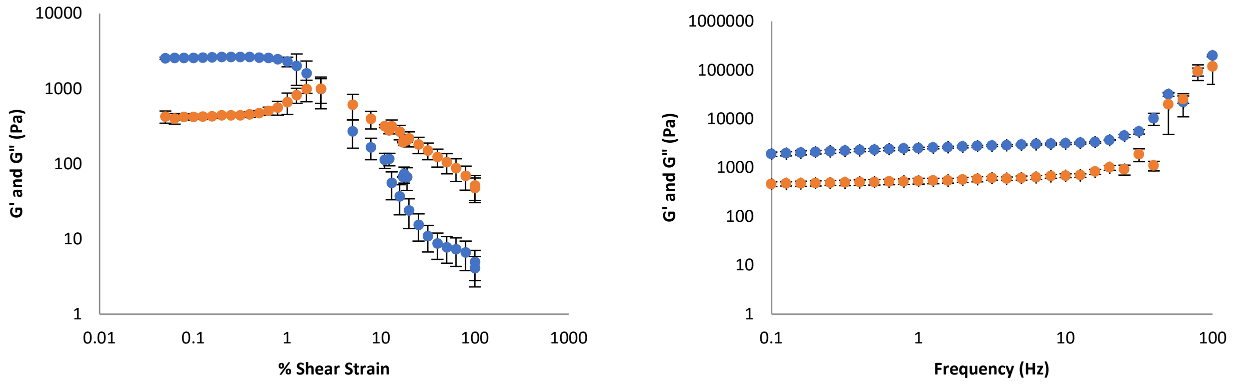

Figure S29. Elastic (G’, blue circles) and viscous (G”, orange circles) moduli of alginate hydrogel (0.8% wt/vol) with increasing shear strain (left) and frequency (right).

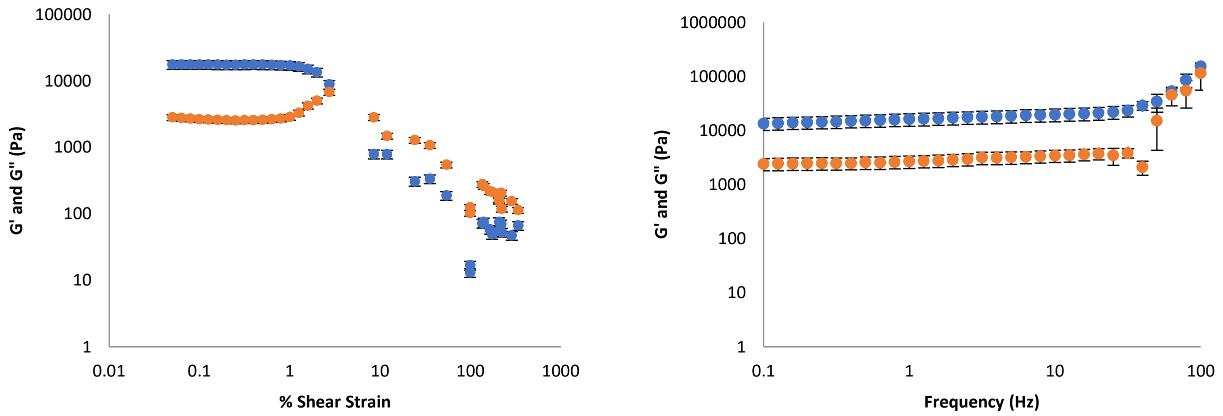

Figure S30. Elastic (G’, blue circles) and viscous (G”, orange circles) moduli of alginate hydrogel (1.3 % wt/vol) with increasing shear strain (left) and frequency (right).

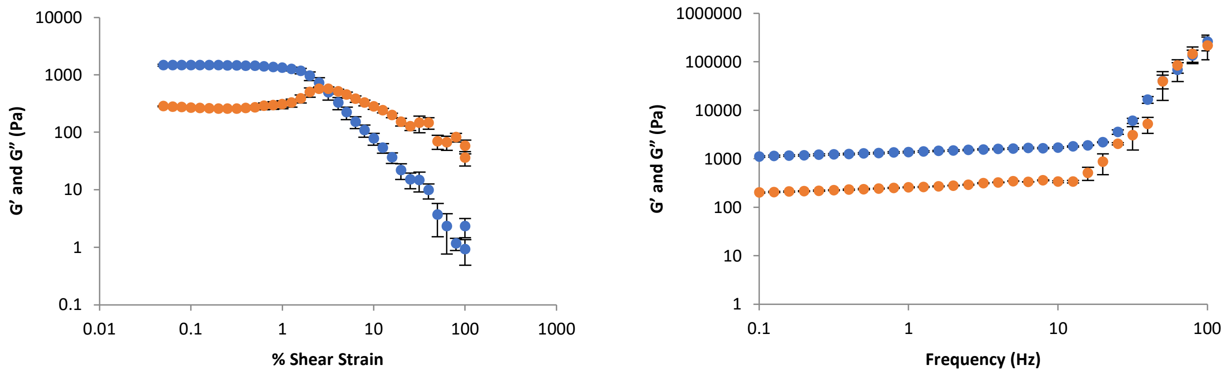

Figure S31. Elastic (G’, blue circles) and viscous (G”, orange circles) moduli of alginate hydrogel (1.3 % wt/vol) loaded with rosuvastatin with increasing shear strain (left) and frequency (right).

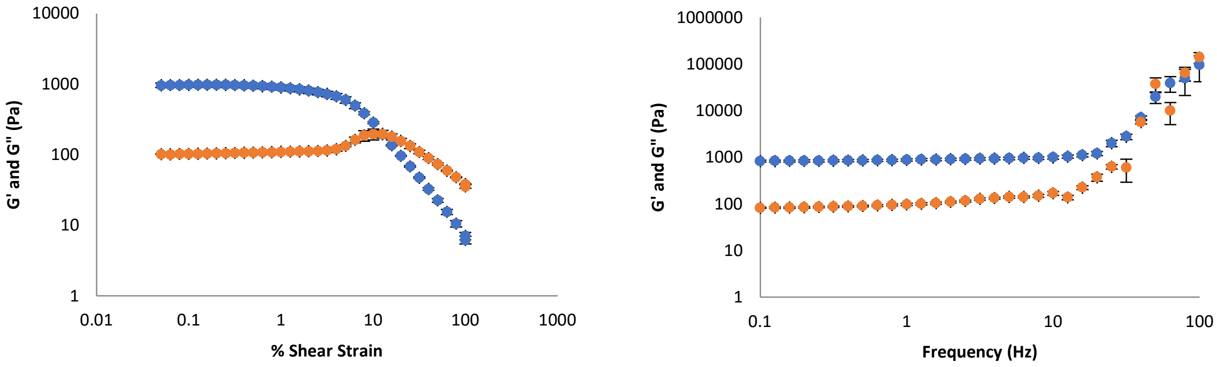

Figure S32. Elastic (G’, blue circles) and viscous (G”, orange circles) moduli of DBS-COOH/alginate two-component hydrogel (0.3% wt/vol DBS-COOH and 0.1% wt/vol alginate) with increasing shear strain (left) and frequency (right).

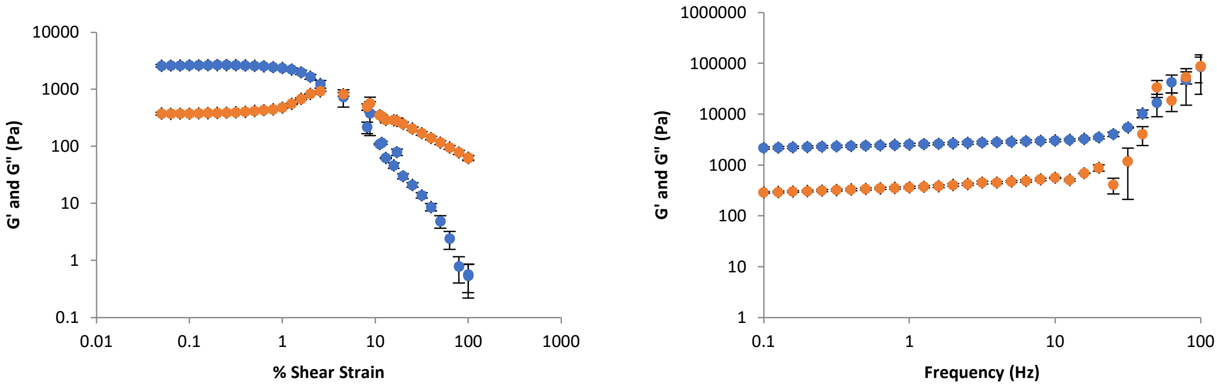

Figure S33. Elastic (G’, blue circles) and viscous (G”, orange circles) moduli of DBS-COOH/alginate two-component hydrogel (0.3% wt/vol of both gelators) with increasing shear strain (left) and frequency (right).

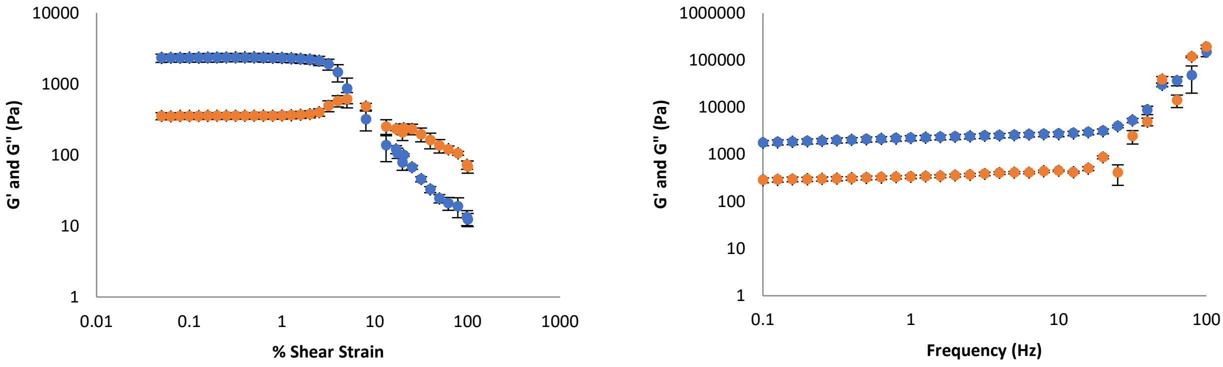

Figure S34. Elastic (G’, blue circles) and viscous (G”, orange circles) moduli of DBS-COOH/alginate two-component hydrogel (0.3% wt/vol DBS-COOH and 0.5% wt/vol alginate) with increasing shear strain (left) and frequency (right).

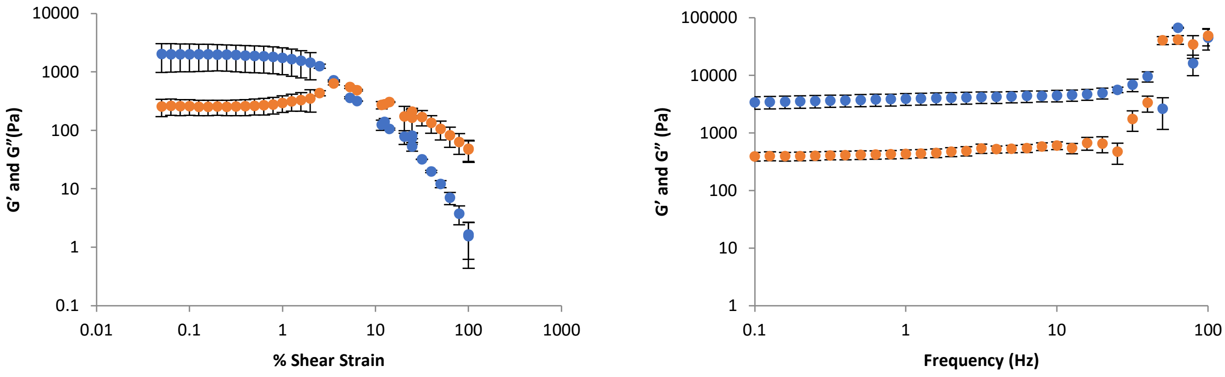

Figure S35. Elastic (G’, blue circles) and viscous (G”, orange circles) moduli of DBS-COOH/alginate two-component hydrogel (0.3% wt/vol DBS-COOH and 0.5% wt/vol alginate) loaded with rosuvastatin, with increasing shear strain (left) and frequency (right).

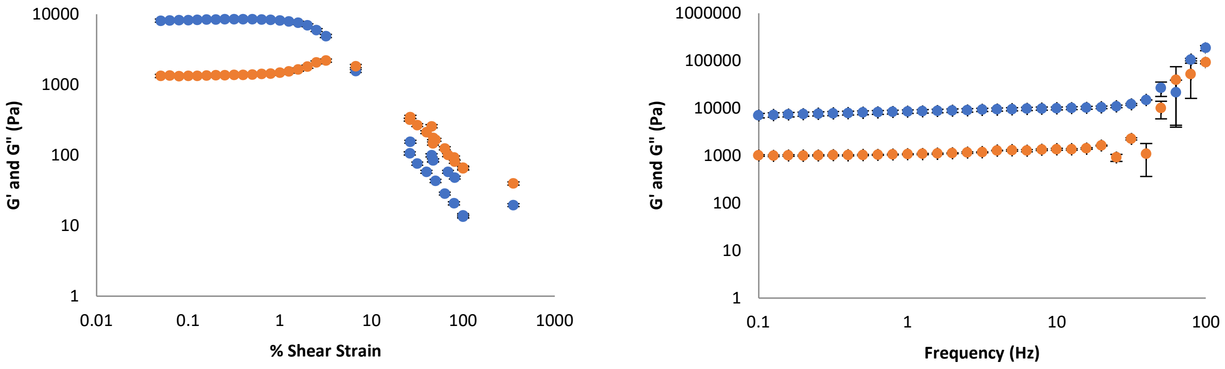

Figure S36. Elastic (G’, blue circles) and viscous (G”, orange circles) moduli of DBS-COOH/alginate two-component hydrogel (0.3% wt/vol DBS-COOH and 1.0% wt/vol alginate) with increasing shear strain (left) and frequency (right).

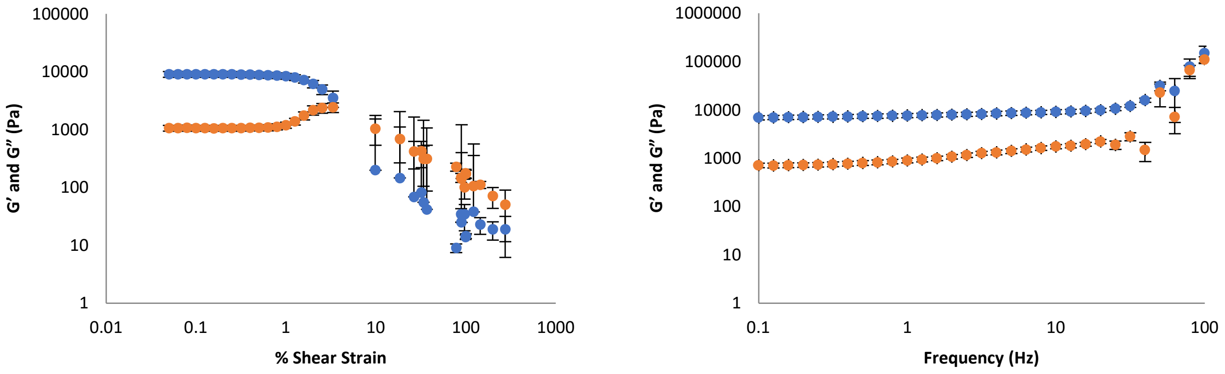

Figure S37. Elastic (G’, blue circles) and viscous (G”, orange circles) moduli of DBS-CONHNH$_2$/alginate two-component hydrogel (0.3% wt/vol DBS-CONHNH$_2$ and 0.5% wt/vol alginate) with increasing shear strain (left) and frequency (right).

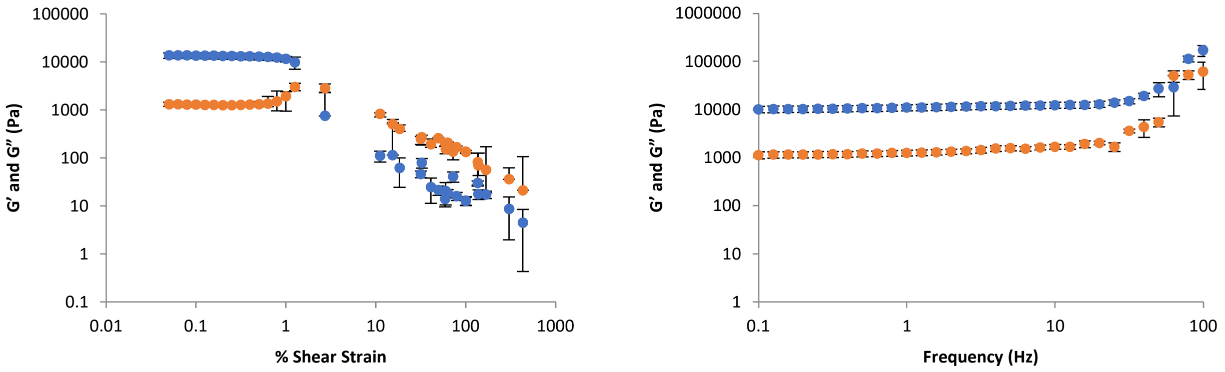

Figure S38. Elastic (G’, blue circles) and viscous (G”, orange circles) moduli of DBS-CONHNH$_2$/alginate two-component hydrogel (0.3% wt/vol DBS-CONHNH$_2$ and 0.5% wt/vol alginate) loaded with rosuvastatin, with increasing shear strain (left) and frequency (right).

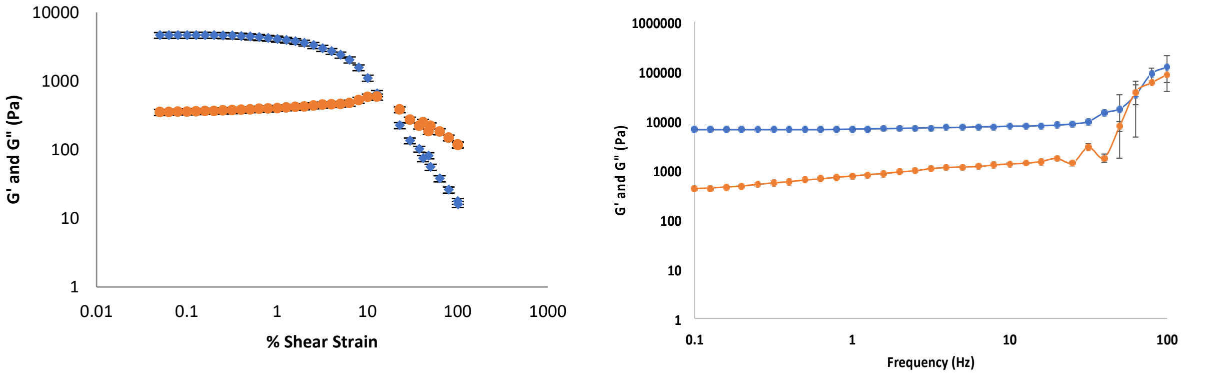

Figure S39. Elastic (G’, blue circles) and viscous (G”, orange circles) moduli of DBS-COOH/DBS-CONHNH$_2$ hydrogel (0.3% wt/vol DBS-COOH and 0.3% wt/vol DBS-CONHNH$_2$) with increasing shear strain (left) and frequency (right).

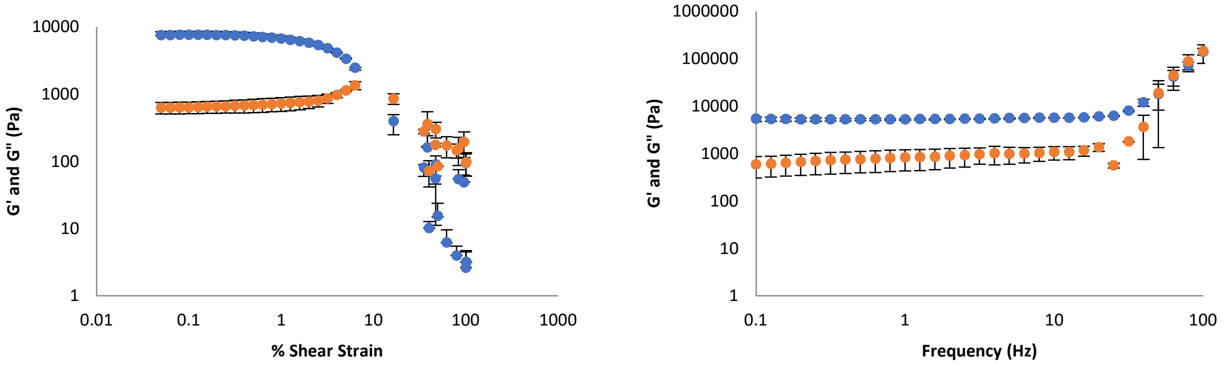

Figure S40. Elastic (G’, blue circles) and viscous (G”, orange circles) moduli of DBS-COOH/DBS-CONHNH$_2$/alginate hydrogel (0.3% wt/vol both LMWGs and 0.1% wt/vol alginate) with increasing shear strain (left) and frequency (right).

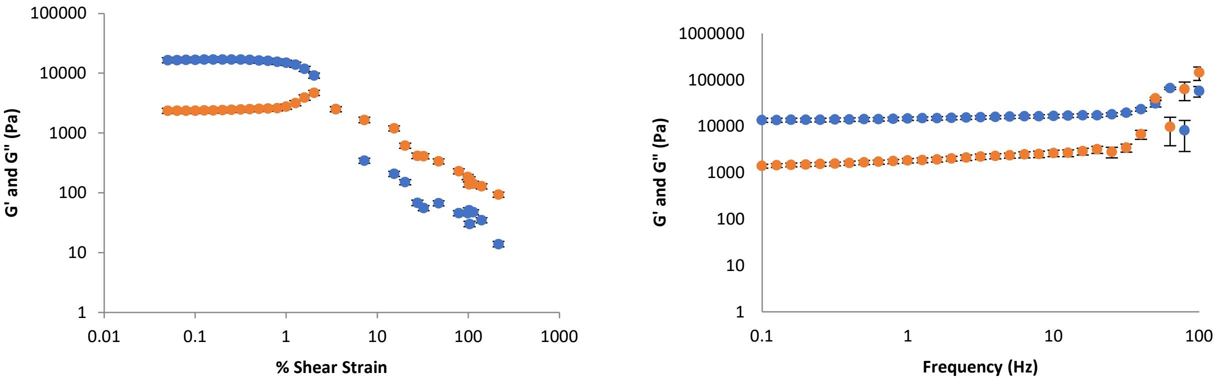

Figure S41. Elastic (G’, blue circles) and viscous (G”, orange circles) moduli of DBS-COOH/DBS-CONHNH$_2$/alginate multicomponent hydrogel (0.3% wt/vol of all gelators) with increasing shear strain (left) and frequency (right).

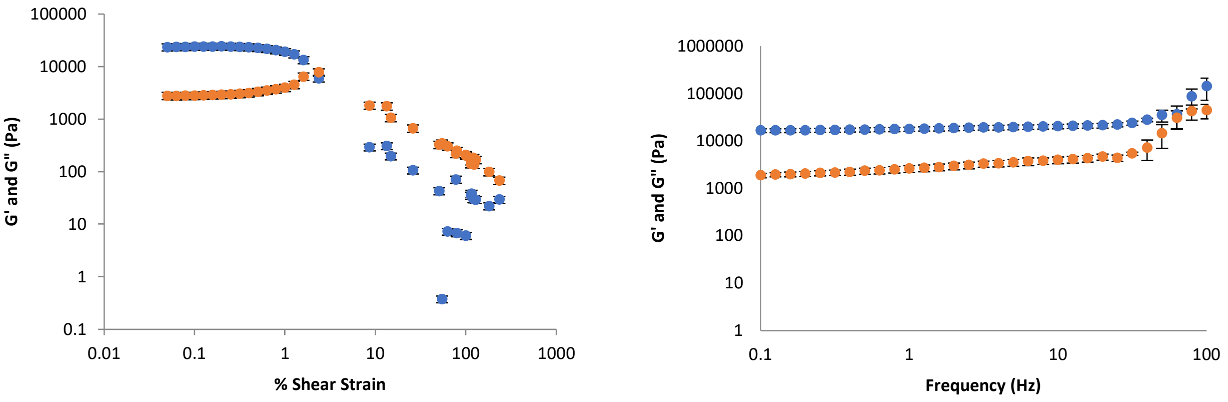

Figure S42. Elastic (G’, blue circles) and viscous (G”, orange circles) moduli of DBS-COOH/DBS-CONHNH$_2$/alginate hydrogel (0.3% wt/vol both LMWGs and 0.5% wt/vol alginate) with increasing shear strain (left) and frequency (right).

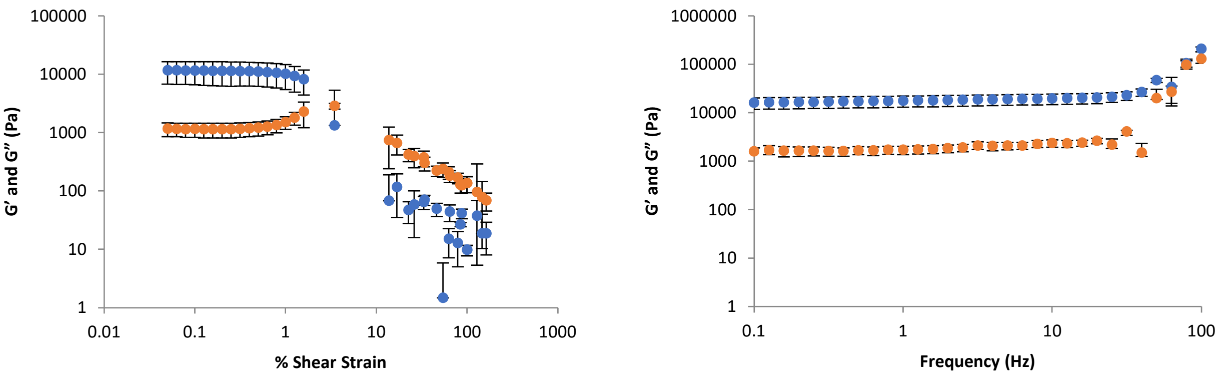

Figure S43. Elastic (G’, blue circles) and viscous (G”, orange circles) moduli of DBS-COOH/DBS-CONHNH$_2$/alginate hydrogel (0.3% wt/vol both LMWGs and 0.5% wt/vol alginate) loaded with rosuvastatin, with increasing shear strain (left) and frequency (right).

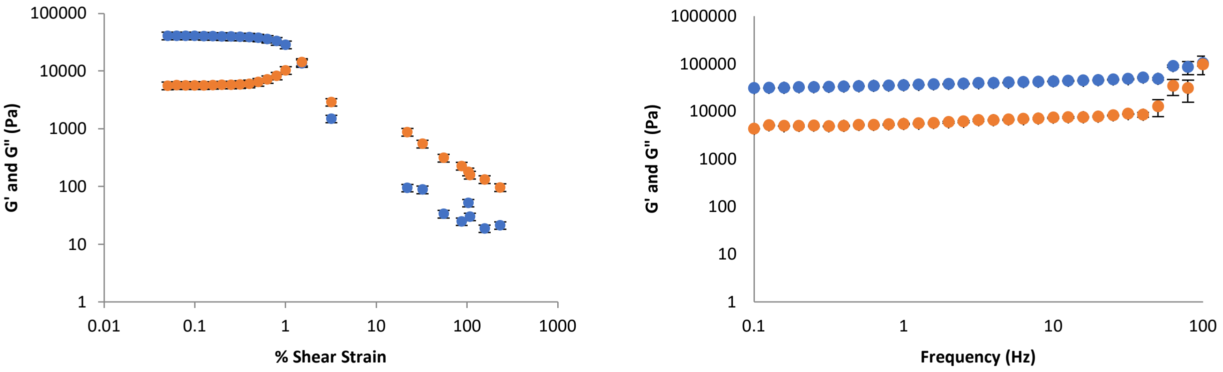

Figure S44. Elastic (G’, blue circles) and viscous (G”, orange circles) moduli of DBS-COOH/DBS-CONHNH$_2$/alginate hydrogel (0.3% wt/vol both LMWGs and 1.0% wt/vol alginate) with increasing shear strain (left) and frequency (right).

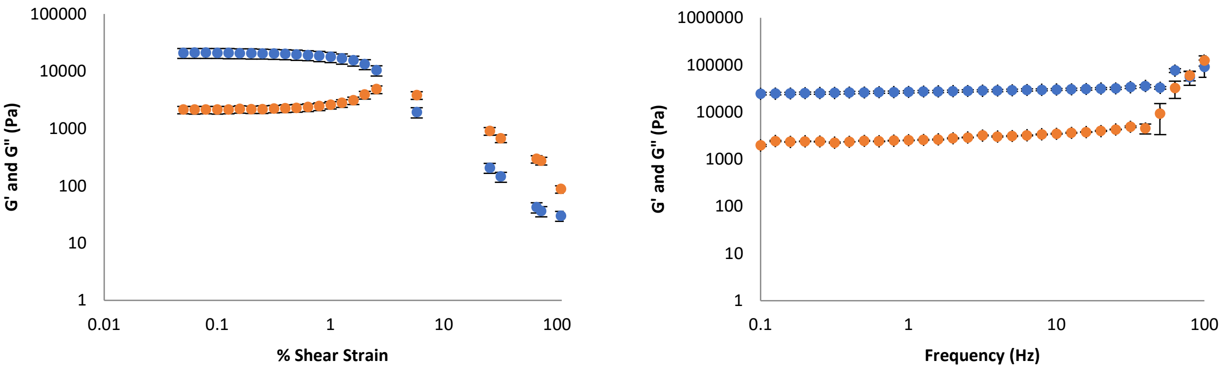

Figure S45. Elastic (G’, blue circles) and viscous (G”, orange circles) moduli of DBS-COOH/DBS-CONHNH$_2$/alginate hydrogel (0.3% wt/vol both LMWGs and 1.0% wt/vol alginate) with increasing shear strain (left) and frequency (right), after exposure to NaOH (0.5 M, 60 µL).

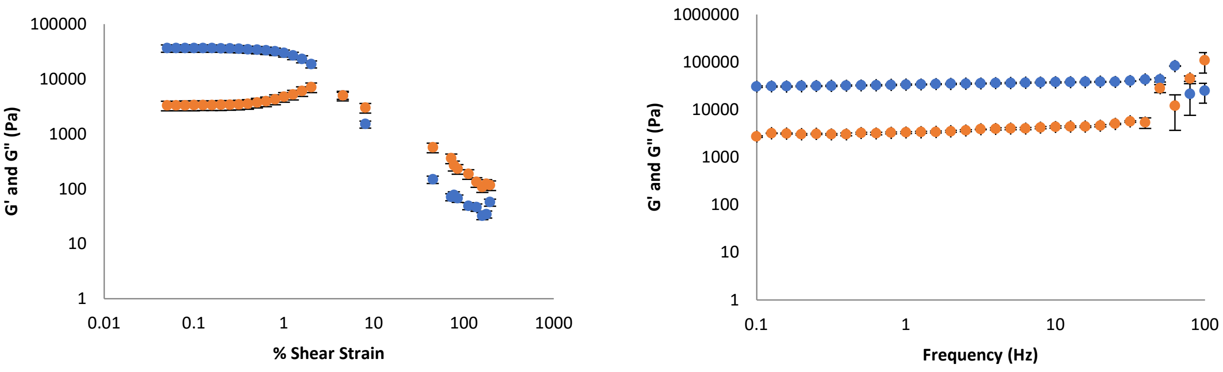

Figure S46. Elastic (G’, blue circles) and viscous (G”, orange circles) moduli of DBS-COOH/DBS-CONHNH$_2$/alginate hydrogel (0.3% wt/vol both LMWGs and 1.0% wt/vol alginate) with increasing shear strain (left) and frequency (right), after exposure to NaOH (0.5 M, 60 µL) and subsequent addition of GdL.

S10 Drug Release Studies

S10.1 Release assay. The gels for rosuvastatin calcium release studies were prepared as described in Section S2. These were loaded with rosuvastatin calcium by adding to each sample a 0.11 mM rosuvastatin calcium aqueous solution (4 mL). After 24 hours, the drug solution was removed and used to quantify the exact amount of drug incorporated into each gel by UV-vis spectroscopy (c.a. 0.22 μmol/gel). 10 mM Tris-HCl/150 mM NaCl (pH 7.4) or sodium acetate buffer (0.1 M - pH 4.0) was then added to each sample (6 mL) as the release medium. The samples were incubated at 37 °C for the duration of the study (24 hours). At regular time intervals, 2 mL aliquots of each sample were transferred into a cuvette and the UV-vis absorbance at 251 nm was monitored before the aliquot was returned to the sample. To ensure reproducibility, the data were collected in triplicate (or duplicate) and the average is shown. Control experiments with gels containing no rosuvastatin calcium were also carried out. A calibration curve of rosuvastatin calcium was obtained in 10 mM Tris-HCl/150 mM NaCl and 0.1 M sodium acetate buffer to quantify the exact amount of drug released.

Table S3. Rosuvastatin calcium release in 10 mM Tris-HCl/150 mM NaCl from gels prepared in sample vials.

Time (hours)	Rosuvastatin calcium (%) released from hydrogels in 10 mM Tris-HCl/150 mM NaCl buffer						
	Alginate	DBS-CONHNH$_2$	DBS-CONHNH$_2$/Alginate	DBS-COOH	DBS-COOH/Alginate	DBS-CONHNH$_2$/DBS-COOH	DBS-CONHNH$_2$/DBS-COOH/Alginate
0	0.00	0.00	0.00	0.00	0.00	0.00	0.00
0.5	31.89	-	9.47	-	37.60	40.54	11.82
1	33.46	4.16	21.05	3.76	51.95	53.72	25.85
1.5	37.39	8.46	25.18	29.19	62.90	63.97	38.88
2	40.18	13.71	27.13	43.76	68.23	68.43	51.30
2.5	40.68	15.23	33.03	59.19	76.03	78.77	58.34
3	41.21	19.26	36.81	64.35	81.14	77.07	65.87
4	42.82	22.99	39.96	82.85	89.99	82.44	76.02
5	41.65	27.28	45.07	84.24	89.23	87.62	79.47
6	40.67	30.12	44.85	84.43	88.82	95.16	78.96
7	42.30	30.42	45.02	100.00	90.93	93.09	84.86
8	-	34.77	44.21	98.18	-	-	-
24	42.01	39.14	39.85	100.00	99.65	86.33	95.52

Table S4. Rosuvastatin calcium release in 10 mM Tris-HCl/150 mM NaCl from gel beads.

Time (hours)	Rosuvastatin calcium (%) released from gel beads in 10 mM Tris-HCl/150 mM NaCl buffer			
	Alginate gel beads	DBS-CONHNH$_2$/Alginate gel beads	DBS-COOH/Alginate gel beads	DBS-CONHNH$_2$/DBS-COOH/Alginate gel beads
0	0.00	0.00	0.00	0.00
0.5	30.46	37.95	47.06	3.80
1	37.33	46.99	68.28	11.63
1.5	38.76	55.62	78.79	17.41
2	40.14	59.38	81.02	17.67
2.5	42.77	59.08	84.17	17.47
3	42.23	58.99	84.43	20.41
4	41.27	60.43	83.84	20.69
5	42.88	60.86	85.02	21.72
6	42.88	59.80	83.46	24.47
7	40.97	57.09	82.74	-
8	41.39	56.69	-	26.66
24	41.51	53.01	80.85	31.68
48	45.62	54.67	89.66	37.23
72	46.49	58.76	91.38	46.16
7 days	46.41	65.88	87.08	45.94

Table S5. Rosuvastatin calcium release in 0.1 M sodium acetate buffer from gels prepared in sample vials.

Time (hours)	Rosuvastatin calcium (%) released from hydrogels in 0.1 M Sodium acetate buffer						
	Alginate	DBS-CONHNH$_2$	DBS-CONHNH$_2$/Alginate	DBS-COOH	DBS-COOH/Alginate	DBS-CONHNH$_2$/DBS-COOH	DBS-CONHNH$_2$/DBS-COOH/Alginate
0	0.00	0.00	0.00	0.00	0.00	0.00	0.00
0.5	18.63	6.47	-	11.26	16.06	21.35	25.39
1	29.68	7.56	12.02	18.49	27.80	34.39	47.49
1.5	33.05	11.36	21.20	21.10	32.31	37.81	51.70
2	36.99	19.45	25.10	19.41	36.22	36.13	56.38
2.5	36.91	18.24	33.28	19.08	37.68	34.43	58.74
3	37.04	19.57	35.34	26.59	38.42	36.74	57.99
4	39.55	26.45	46.32	18.64	41.03	37.11	57.33
5	38.82	27.14	50.12	19.97	41.27	38.96	52.37
6	38.00	27.06	52.45	22.95	40.50	34.90	47.40
7	38.52	29.83	51.75	16.07	39.64	37.78	43.88
8	37.98	28.78	-	21.75	41.37	36.52	43.99
24	38.79	21.06	44.13	29.28	40.88	32.82	35.58

Table S6. Rosuvastatin calcium release in 0.1 M sodium acetate buffer from gel beads.

Time (hours)	Rosuvastatin calcium (%) released from hydrogels in 0.1 M Sodium acetate buffer			
	Alginate gel beads	DBS-CONHNH$_2$/Alginate gel beads	DBS-COOH/Alginate gel beads	DBS-CONHNH$_2$/DBS-COOH/Alginate gel beads
0	0.00	0.00	0.00	0.00
0.5	24.55	31.76	30.60	13.94
1	28.23	30.14	38.42	1.47
1.5	29.49	28.05	45.41	2.90
2	29.33	31.43	46.25	2.58
2.5	30.31	26.79	45.69	4.07
3	29.41	27.32	44.53	5.33
4	31.70	30.37	46.30	3.24
5	33.29	32.20	47.93	5.91
6	32.15	28.77	47.71	3.73
7	30.75	-	44.68	5.00
8	30.63	26.32	48.33	6.44
24	33.01	29.52	44.21	7.27
48	39.08	35.12	49.35	11.63
72	35.06	35.58	47.77	9.36
7 days	35.24	49.37	42.62	9.31

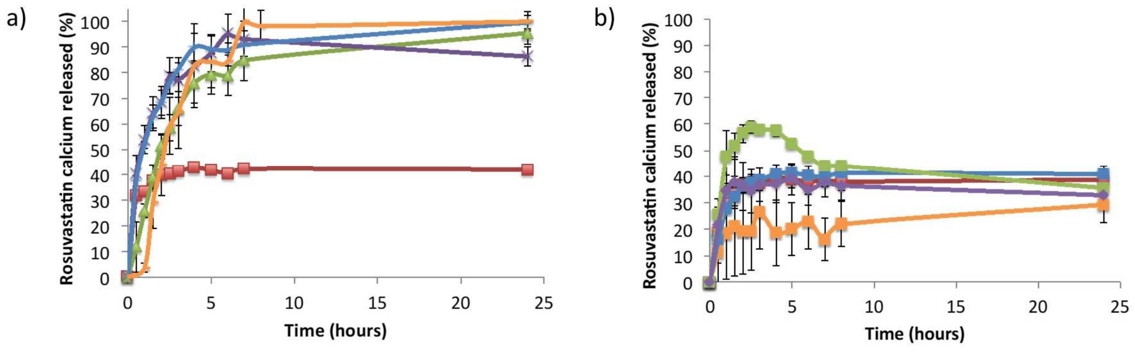

Fig. S47. Rosuvastatin calcium % released from gels prepared in sample vials in (a) 10 mM Tris-HCl/150 mM NaCl and (b) 0.1 M sodium acetate. Gels: alginate (red line), DBS-COOH (orange line), DBS-COOH/DBS-CONHNH$_2$ (purple line), DBS-COOH/alginate (blue line), DBS-COOH/DBS-CONHNH$_2$/alginate (green line).

Table S7. Rosuvastatin calcium release in NaCl/HCl buffer (pH 1.2) from gel beads.

Time (hours)	Rosuvastatin calcium (%) released from gel beads in NaCl/HCl buffer at pH 1.2			
	Alginate gel beads	DBS-CONHNH$_2$/Alginate gel beads (some bead degradation)	DBS-COOH/Alginate gel beads	DBS-CONHNH$_2$/DBS-COOH/Alginate gel beads (some bead degradation)
0	0	0.00	0.00	0.00
0.5	14.86	31.44	13.92	24.63
1	19.44	42.14	17.93	59.22
2	17.45	54.04	25.55	78.65
3	18.59	63.09	25.42	69.32
4	17.89	72.31	37.53	50.56
5	15.89	61.81	33.86	55.20
6	16.03	63.03	36.94	57.63
7	15.92	66.85	39.59	47.20
8	16.25	62.24	37.22	45.83
24	22.09	66.73	35.89	55.58
48	22.32	67.01	39.26	53.75
72	24.52	63.45	43.70	61.58
7 days	24.28	64.76	44.26	55.42

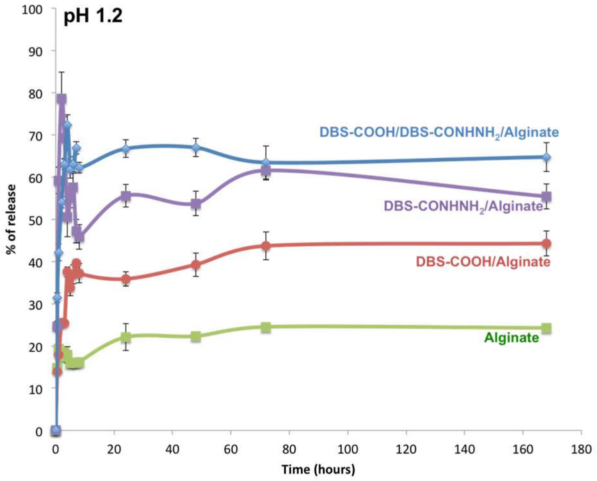

Figure S48. Rosuvastatin calcium % released in NaCl/HCl buffer (pH 1.2) from gel beads – it should be noted that for gel beads including DBS-CONHNH$_2$, some degradation of gel beads was observed.

S12 References

[1] B. O. Okesola, D. K. Smith, *Chem. Commun.*, **2013**, *49*, 11164-11166.

[2] D. J. Cornwell, B. O. Okesola, D. K. Smith, *Soft Matter*, **2013**, *9*, 8730-8736.

[3] C. C. Piras, D. K. Smith, *Chem. Eur. J.* **2019,** *25*, 11318-11326
